# The role of sexually transmitted infections in male circumcision effectiveness against HIV – insights from clinical trial simulation

**DOI:** 10.1186/1742-7622-3-19

**Published:** 2006-12-22

**Authors:** Kamal Desai, Marie-Claude Boily, Geoff P Garnett, Benoît R Mâsse, Stephen Moses, Robert C Bailey

**Affiliations:** 1 Department of Infectious Disease Epidemiology, Imperial College London, St-Mary's Hospital, Norfolk Place, London, W2 1PG, UK; 2 Statistical Center for HIV/AIDS Research & Prevention, Fred Hutchinson Cancer Research Center, Seattle, WA, USA; 3 Department of Medical Microbiology, Community Health Sciences and Medicine, University of Manitoba, Winnipeg, Canada; 4 Division of Epidemiology, University of Illinois at Chicago, Chicago, IL, USA

## Abstract

**Background:**

A landmark randomised trial of male circumcision (MC) in Orange Farm, South Africa recently showed a large and significant reduction in risk of HIV infection, reporting MC effectiveness of 61% (95% CI: 34%–77%). Additionally, two further randomised trials of MC in Kisumu, Kenya and Rakai, Uganda were recently stopped early to report 53% and 48% effectiveness, respectively. Since MC may protect against both HIV and certain sexually transmitted infections (STI), which are themselves cofactors of HIV infection, an important question is the extent to which this estimated effectiveness against HIV is mediated by the protective effect of circumcision against STI. The answer lies in the trial data if the appropriate statistical analyses can be identified to estimate the separate efficacies against HIV and STI, which combine to determine overall effectiveness.

**Objectives and Methods:**

Focusing on the MC trial in Kisumu, we used a stochastic prevention trial simulator (1) to determine whether statistical analyses can validly estimate efficacy, (2) to determine whether MC efficacy against STI alone can produce large effectiveness against HIV and (3) to estimate the fraction of all HIV infections prevented that are attributable to efficacy against STI when both efficacies combine.

**Results:**

Valid estimation of separate efficacies against HIV and STI as well as MC effectiveness is feasible with available STI and HIV trial data, under Kisumu trial conditions. Under our parameter assumptions, high overall effectiveness of MC against HIV was observed only with a high MC efficacy against HIV and was not possible on the basis of MC efficacy against STI alone. The fraction of all HIV infections prevented which were attributable to MC efficacy against STI was small, except when efficacy of MC specifically against HIV was very low. In the three MC trials which reported between 48% and 61% effectiveness (combining STI and HIV efficacies), the fraction of HIV infections prevented in circumcised males which were attributable to STI was unlikely to be more than 10% to 20%.

**Conclusion:**

Estimation of efficacy, attributable fraction and effectiveness leads to improved understanding of trial results, gives trial results greater external validity and is essential to determine the broader public health impact of circumcision to men and women.

## Background

The role of male circumcision (MC) in protecting against HIV infection in sub-Saharan Africa has been controversial since the beginning of the HIV epidemic. This is because evidence derived from observational studies is prone to bias due to confounding risk factors and because MC as a HIV prevention strategy can be seen as unethical [1-3]. There is mounting epidemiological evidence and plausible biological explanation to indicate that MC can protect against HIV both directly and indirectly [4-11]. Risk due to abrasions suffered by foreskin in uncircumcised men, inflammatory conditions under the foreskin and high concentrations of Langerhans cells which are targets for HIV infection can be directly minimised by MC [4,5]. Evidence also exists for an association between MC and reduced risk of infection with certain sexually transmitted infections (STI) such as chancroid, syphilis, gonorrhoea and HSV [6-12]. Because these STIs are co-factors of HIV transmission [13], indirect protection against HIV, mediated by the benefit of MC against STIs, is also possible.

The role of male circumcision as a HIV prevention tool is now supported by a landmark randomised trial that showed a large and significant effectiveness against HIV (61%; 95% CI: 34%–77%) among a sample of adult men in Orange Farm, South Africa [14]. Recently, two additional independent randomised MC trials in Kisumu, Kenya [15,16] and Rakai, Uganda [17] were stopped early upon finding significant effectiveness against HIV of 53% and 48%, respectively [18]. The magnitude of these estimates is in line with meta-analyses of observational studies [8,19-22]. Now, further efforts are being made to better understand trial results and their implications for public health [23,24]. In particular, the role STIs played in the overall effectiveness of MC against HIV in the three trials needs to be better understood as does the applicability of the trial findings to other settings with different HIV and STI epidemiology. This cannot be ascertained from MC trials alone because they, like most HIV prevention trials, have primary objective to demonstrate the overall reduction in the risk of HIV infection, called effectiveness. While estimation of effectiveness addresses the primary objective of the trial by comparing infection rates between circumcised men and controls, it does not specifically measure the separate effects of MC against HIV and other STIs. Thus, a clinical trial of MC conducted in a setting with high levels of STI may give rise to an important difference in HIV infection between trial arms, pointing to a positive overall effectiveness without pinpointing why this result is achieved. Without this clarity on a positive effectiveness, it is difficult to know to which extent the overall HIV risk reduction is due to the direct versus indirect protective effect of MC against HIV.

These questions can be answered if the appropriate statistical analyses can be identified. This involves estimation of the efficacies of MC against HIV and STI separately, as well as the usual estimation of overall effectiveness from randomised trials. These efficacy and effectiveness estimates can then be combined to deduce the fraction of HIV infections prevented by MC which are attributable to STI.

In this paper we use a prevention trial simulator of circumcision, using data from the Kisumu MC trial, where HIV and STI both circulate (1) to demonstrate which statistical analyses validly estimate efficacy against HIV and STI; (2) to examine whether the efficacy of MC on STI alone can result in a large overall estimates of HIV effectiveness a in randomised trial when MC does not have a direct protective effect against HIV; and (3) to examine when both efficacies combine, what is the attributable fraction of all HIV infections prevented by efficacy against STI in a MC trial. The results of our modelling study will facilitate and improve interpretation of the results of the three MC trials. In turn, this will eventually lead to better understanding of the population-level effectiveness and cost-effectiveness of circumcision in settings with different STI and HIV epidemiology.

## Analysis

### Definitions of efficacy, effectiveness and attributable fraction

It is crucial to distinguish between efficacy and effectiveness as they are used in phase 3 HIV prevention trials. Effectiveness, denoted *F*, estimates the total difference in HIV infection rates between trial arms. In a phase 3 trial, the estimated effectiveness will depend on several factors. These include the direct reduction in female-to-male HIV transmission probability due to circumcision, the direct reduction in female-to-male STI transmission, prevalence of STI in trial participants and their partners, and strength of the HIV-STI epidemiological interaction. Since STI prevalence can change with time in trial participants, effectiveness estimates can also depend on the duration of follow-up. Thus, effectiveness is an estimate of the aggregate effects of several factors whose interpretation is limited to the trial context in which it is estimated and is a time-dependent quantity. We use the term effectiveness in its clinical trial sense which is more limited than its epidemiological sense where it would represent impact of MC in a general population. In particular, the effectiveness estimated in a clinical trial says nothing about the herd effect of MC benefiting females.

Efficacy against HIV, denoted *E*_*H*_, represents the direct reduction in female-to-male HIV transmission probability due to circumcision, just one of the factors determining effectiveness. Similarly, efficacy against STI, denoted *E*_*S*_, represents the reduction in female-to-male STI transmission probability. Thus, efficacy gives a more 'canonical' description of the effect of circumcision which is independent of setting and time and has a biological interpretation which is more generalisable and simpler to interpret than effectiveness.

We also define attributable fraction, denoted *AF*, as the proportion of all HIV infections prevented that are mediated by MC efficacy against STI. This quantity describes how much of the overall effectiveness of MC against HIV is due to the indirect effect mediated by MC efficacy against STI. The *AF *can be approximated using estimates of *F *and *E*_*H *_(see later).

### Clinical trial simulator

Dynamic stochastic models simulating trial designs are well suited to study this type of question and have been used successfully in the past to evaluate the quality of epidemiological studies on the STI and HIV interaction and to validate statistical analyses for HIV vaccine trials [25-29]. Our stochastic compartmental model (see Endnote) simulates transmission of HIV and one STI in a heterosexual population in which a clinical trial is embedded and is based on previous models that we have used [27,29]. Individuals in the population are heterogenous with respect to sexual activity stratified into classes with different rates of sexual partner acquisition. The STI has been modelled simplistically with two compartments representing infected or not infected. The STI in the model should be thought of as 'generic', rather than representing any one specific STI. HIV infection has been modelled with 5 states representing susceptibles, early- and latent-infection, pre-AIDS and AIDS. Flow of individuals between states is based on flow rates determined from specified parameters or from force of infection equations. Flow rates are instantly updated following each event. Flow of individuals through different HIV states is unidirectional, whereas individuals can recover from STI and be reinfected. Infection rates for different individuals in the population depend on their sexual activity, their HIV or STI infection status, HIV and STI prevalence in their partners, and strength of HIV-STI interaction as cofactors of transmission and circumcision status. Individuals are recruited from the general population for the clinical trial and are followed-up recording the times of their infection events.

The model generates simulated trial data which are then analysed to estimate efficacy against HIV, STI and overall effectiveness against HIV. The efficacy estimates are valid when they agree with the true values embedded in the simulation.

### Model parameters and description of Kisumu circumcision study

The base-case model parameter values were selected according to trial design parameters from the UNIM circumcision trial in Kisumu District of Kenya [15,16], were estimated from the trial's baseline and follow-up data, or were based on relevant published literature. Parameter values and references are given in Table 1.

**Table 1:** Model Parameters. Base-case parameter values used in model simulations and base-case outputs for HIV and STI prevalence and incidence are given. Upper and lower limits for parameters varied in sensitivity analyses are also given.

**Symbol**	**Description of epidemiological parameters**	**Parameter value**	**References**
*γ*_1_,...,*γ*_3_	Rate of progression between 3 stages of HIV infection. Duration of stage 1 is 1/*γ*_1 _= 4.4 months, stage 2 is 1/*γ*_2 _= 6.5 yrs, stage 3 is 1/*γ*_3 _= 2 yrs.	*γ*_1 _= 2.75 *γ*_2 _= 0.154 *γ*_3 _= 0.5	[27,34-41]
βk∗,j,ip MathType@MTEF@5@5@+=feaafiart1ev1aaatCvAUfKttLearuWrP9MDH5MBPbIqV92AaeXatLxBI9gBaebbnrfifHhDYfgasaacH8akY=wiFfYdH8Gipec8Eeeu0xXdbba9frFj0=OqFfea0dXdd9vqai=hGuQ8kuc9pgc9s8qqaq=dirpe0xb9q8qiLsFr0=vr0=vr0dc8meaabaqaciaacaGaaeqabaqabeGadaaakeaaiiGacqWFYoGydaqhaaWcbaGaem4AaS2aaWbaaWqabeaacqGHxiIkaaWccqGGSaalcqWGQbGAcqGGSaalcqWGPbqAaeaacqWGWbaCaaaaaa@36E9@	Per partnership probability of transmission of HIV from individual of sex k*, sexual activity class j and infection phase p (p = 1,...,3) to partner of opposite sex k and class i. Values given are transmission probabilities for females (k* = 1) of low activity class (j = 1,2 = lowf) or high activity class (j = 3,...,6 = highf) to males of low activity class (i = 1,2 = lowm) or high activity class (i = 3,...,6 = highm). Transmission probabilities for male to female are double of these below. Under sensitivity analyses, values were multiplied by a factor of 0.5, 2.0 or 4.0.		
	β1,lowf,lowm1 MathType@MTEF@5@5@+=feaafiart1ev1aaatCvAUfKttLearuWrP9MDH5MBPbIqV92AaeXatLxBI9gBaebbnrfifHhDYfgasaacH8akY=wiFfYdH8Gipec8Eeeu0xXdbba9frFj0=OqFfea0dXdd9vqai=hGuQ8kuc9pgc9s8qqaq=dirpe0xb9q8qiLsFr0=vr0=vr0dc8meaabaqaciaacaGaaeqabaqabeGadaaakeaaiiGacqWFYoGydaqhaaWcbaGaeGymaeJaeiilaWIaemiBaWMaem4Ba8Maem4DaCNaemOzayMaeiilaWIaemiBaWMaem4Ba8Maem4DaCNaemyBa0gabaGaeGymaedaaaaa@3D57@ = 0.08 β1,lowf,lowm2 MathType@MTEF@5@5@+=feaafiart1ev1aaatCvAUfKttLearuWrP9MDH5MBPbIqV92AaeXatLxBI9gBaebbnrfifHhDYfgasaacH8akY=wiFfYdH8Gipec8Eeeu0xXdbba9frFj0=OqFfea0dXdd9vqai=hGuQ8kuc9pgc9s8qqaq=dirpe0xb9q8qiLsFr0=vr0=vr0dc8meaabaqaciaacaGaaeqabaqabeGadaaakeaaiiGacqWFYoGydaqhaaWcbaGaeGymaeJaeiilaWIaemiBaWMaem4Ba8Maem4DaCNaemOzayMaeiilaWIaemiBaWMaem4Ba8Maem4DaCNaemyBa0gabaGaeGOmaidaaaaa@3D59@ = 0.0032 β1,lowf,highm1 MathType@MTEF@5@5@+=feaafiart1ev1aaatCvAUfKttLearuWrP9MDH5MBPbIqV92AaeXatLxBI9gBaebbnrfifHhDYfgasaacH8akY=wiFfYdH8Gipec8Eeeu0xXdbba9frFj0=OqFfea0dXdd9vqai=hGuQ8kuc9pgc9s8qqaq=dirpe0xb9q8qiLsFr0=vr0=vr0dc8meaabaqaciaacaGaaeqabaqabeGadaaakeaaiiGacqWFYoGydaqhaaWcbaGaeGymaeJaeiilaWIaemiBaWMaem4Ba8Maem4DaCNaemOzayMaeiilaWIaemiAaGMaemyAaKMaem4zaCMaemiAaGMaemyBa0gabaGaeGymaedaaaaa@3E7C@ = 0.043 β1,lowf,highm2 MathType@MTEF@5@5@+=feaafiart1ev1aaatCvAUfKttLearuWrP9MDH5MBPbIqV92AaeXatLxBI9gBaebbnrfifHhDYfgasaacH8akY=wiFfYdH8Gipec8Eeeu0xXdbba9frFj0=OqFfea0dXdd9vqai=hGuQ8kuc9pgc9s8qqaq=dirpe0xb9q8qiLsFr0=vr0=vr0dc8meaabaqaciaacaGaaeqabaqabeGadaaakeaaiiGacqWFYoGydaqhaaWcbaGaeGymaeJaeiilaWIaemiBaWMaem4Ba8Maem4DaCNaemOzayMaeiilaWIaemiAaGMaemyAaKMaem4zaCMaemiAaGMaemyBa0gabaGaeGOmaidaaaaa@3E7E@ = 0.0017 β1,highf,lowm1 MathType@MTEF@5@5@+=feaafiart1ev1aaatCvAUfKttLearuWrP9MDH5MBPbIqV92AaeXatLxBI9gBaebbnrfifHhDYfgasaacH8akY=wiFfYdH8Gipec8Eeeu0xXdbba9frFj0=OqFfea0dXdd9vqai=hGuQ8kuc9pgc9s8qqaq=dirpe0xb9q8qiLsFr0=vr0=vr0dc8meaabaqaciaacaGaaeqabaqabeGadaaakeaaiiGacqWFYoGydaqhaaWcbaGaeGymaeJaeiilaWIaemiAaGMaemyAaKMaem4zaCMaemiAaGMaemOzayMaeiilaWIaemiBaWMaem4Ba8Maem4DaCNaemyBa0gabaGaeGymaedaaaaa@3E7C@ = 0.025 β1,highf,lowm2 MathType@MTEF@5@5@+=feaafiart1ev1aaatCvAUfKttLearuWrP9MDH5MBPbIqV92AaeXatLxBI9gBaebbnrfifHhDYfgasaacH8akY=wiFfYdH8Gipec8Eeeu0xXdbba9frFj0=OqFfea0dXdd9vqai=hGuQ8kuc9pgc9s8qqaq=dirpe0xb9q8qiLsFr0=vr0=vr0dc8meaabaqaciaacaGaaeqabaqabeGadaaakeaaiiGacqWFYoGydaqhaaWcbaGaeGymaeJaeiilaWIaemiAaGMaemyAaKMaem4zaCMaemiAaGMaemOzayMaeiilaWIaemiBaWMaem4Ba8Maem4DaCNaemyBa0gabaGaeGOmaidaaaaa@3E7E@ = 0.001 β1,highf,highm1 MathType@MTEF@5@5@+=feaafiart1ev1aaatCvAUfKttLearuWrP9MDH5MBPbIqV92AaeXatLxBI9gBaebbnrfifHhDYfgasaacH8akY=wiFfYdH8Gipec8Eeeu0xXdbba9frFj0=OqFfea0dXdd9vqai=hGuQ8kuc9pgc9s8qqaq=dirpe0xb9q8qiLsFr0=vr0=vr0dc8meaabaqaciaacaGaaeqabaqabeGadaaakeaaiiGacqWFYoGydaqhaaWcbaGaeGymaeJaeiilaWIaemiAaGMaemyAaKMaem4zaCMaemiAaGMaemOzayMaeiilaWIaemiAaGMaemyAaKMaem4zaCMaemiAaGMaemyBa0gabaGaeGymaedaaaaa@3FA1@ = 0.025 β1,highf,highm2 MathType@MTEF@5@5@+=feaafiart1ev1aaatCvAUfKttLearuWrP9MDH5MBPbIqV92AaeXatLxBI9gBaebbnrfifHhDYfgasaacH8akY=wiFfYdH8Gipec8Eeeu0xXdbba9frFj0=OqFfea0dXdd9vqai=hGuQ8kuc9pgc9s8qqaq=dirpe0xb9q8qiLsFr0=vr0=vr0dc8meaabaqaciaacaGaaeqabaqabeGadaaakeaaiiGacqWFYoGydaqhaaWcbaGaeGymaeJaeiilaWIaemiAaGMaemyAaKMaem4zaCMaemiAaGMaemOzayMaeiilaWIaemiAaGMaemyAaKMaem4zaCMaemiAaGMaemyBa0gabaGaeGOmaidaaaaa@3FA3@ = 0.001	β1,lowf,lowm3 MathType@MTEF@5@5@+=feaafiart1ev1aaatCvAUfKttLearuWrP9MDH5MBPbIqV92AaeXatLxBI9gBaebbnrfifHhDYfgasaacH8akY=wiFfYdH8Gipec8Eeeu0xXdbba9frFj0=OqFfea0dXdd9vqai=hGuQ8kuc9pgc9s8qqaq=dirpe0xb9q8qiLsFr0=vr0=vr0dc8meaabaqaciaacaGaaeqabaqabeGadaaakeaaiiGacqWFYoGydaqhaaWcbaGaeGymaeJaeiilaWIaemiBaWMaem4Ba8Maem4DaCNaemOzayMaeiilaWIaemiBaWMaem4Ba8Maem4DaCNaemyBa0gabaGaeG4mamdaaaaa@3D5B@ = 0.051 β1,lowf,highm3 MathType@MTEF@5@5@+=feaafiart1ev1aaatCvAUfKttLearuWrP9MDH5MBPbIqV92AaeXatLxBI9gBaebbnrfifHhDYfgasaacH8akY=wiFfYdH8Gipec8Eeeu0xXdbba9frFj0=OqFfea0dXdd9vqai=hGuQ8kuc9pgc9s8qqaq=dirpe0xb9q8qiLsFr0=vr0=vr0dc8meaabaqaciaacaGaaeqabaqabeGadaaakeaaiiGacqWFYoGydaqhaaWcbaGaeGymaeJaeiilaWIaemiBaWMaem4Ba8Maem4DaCNaemOzayMaeiilaWIaemiAaGMaemyAaKMaem4zaCMaemiAaGMaemyBa0gabaGaeG4mamdaaaaa@3E80@ = 0.026 β1,highf,lowm3 MathType@MTEF@5@5@+=feaafiart1ev1aaatCvAUfKttLearuWrP9MDH5MBPbIqV92AaeXatLxBI9gBaebbnrfifHhDYfgasaacH8akY=wiFfYdH8Gipec8Eeeu0xXdbba9frFj0=OqFfea0dXdd9vqai=hGuQ8kuc9pgc9s8qqaq=dirpe0xb9q8qiLsFr0=vr0=vr0dc8meaabaqaciaacaGaaeqabaqabeGadaaakeaaiiGacqWFYoGydaqhaaWcbaGaeGymaeJaeiilaWIaemiAaGMaemyAaKMaem4zaCMaemiAaGMaemOzayMaeiilaWIaemiBaWMaem4Ba8Maem4DaCNaemyBa0gabaGaeG4mamdaaaaa@3E80@ = 0.0071 β1,highf,highm3 MathType@MTEF@5@5@+=feaafiart1ev1aaatCvAUfKttLearuWrP9MDH5MBPbIqV92AaeXatLxBI9gBaebbnrfifHhDYfgasaacH8akY=wiFfYdH8Gipec8Eeeu0xXdbba9frFj0=OqFfea0dXdd9vqai=hGuQ8kuc9pgc9s8qqaq=dirpe0xb9q8qiLsFr0=vr0=vr0dc8meaabaqaciaacaGaaeqabaqabeGadaaakeaaiiGacqWFYoGydaqhaaWcbaGaeGymaeJaeiilaWIaemiAaGMaemyAaKMaem4zaCMaemiAaGMaemOzayMaeiilaWIaemiAaGMaemyAaKMaem4zaCMaemiAaGMaemyBa0gabaGaeG4mamdaaaaa@3FA5@ = 0.0071	[27,34,36-28,42-44]
*σ*_*k*,*i*_	Recovery rate of STI infection. Duration of infection is 1/*σ*_*k*,*i *_= 6 months for all sexes k and activity classes i.	*σ*_*k*,*i *_= 2.0. Under sensitivity analyses *σ*_*k*,*i *_= 3.0, 0.75.	[45-47]
*ξ*_*k*,*j*,*i*_	Per partnership probability of transmission of STI from infected individual of sex k and sexual activity class j to partner of opposite sex k* and class i.	*ξ*_*k*,*j*,*i *_= 0.15 for all k,i,j	[45-47]
A_1_, a_2_	Enhancement of HIV transmission given STI infection. a_1 _is the relative risk (multiplicative factor) for increased susceptibility to HIV given STI infection in the partner. a_2 _is the relative risk of increased susceptibility to HIV given STI infection in self.	a_1 _= 4 a_2 _= 3 Under sensitivity analysis a_1 _= 2 & a_2 _= 2 and a_1 _= 6 & a_2 _= 6.	[13]
*α*	Mortality rate due to AIDS	1α MathType@MTEF@5@5@+=feaafiart1ev1aaatCvAUfKttLearuWrP9MDH5MBPbIqV92AaeXatLxBI9gBaebbnrfifHhDYfgasaacH8akY=wiFfYdH8Gipec8Eeeu0xXdbba9frFj0=OqFfea0dXdd9vqai=hGuQ8kuc9pgc9s8qqaq=dirpe0xb9q8qiLsFr0=vr0=vr0dc8meaabaqaciaacaGaaeqabaqabeGadaaakeaadaWccaqaaiabigdaXaqaaGGaciab=f7aHbaaaaa@2F54@ = 1 year	[39-41]
m_k,i_	Annual rate of sexual partner change. m_k,i _is the number of new sexual partners per year for person of sex k and sexual activity class i.	m_k = 1,i = 1...6 _= 1.0 3.5 5 10 50 75 m_k = 2,i = 1...6 _= 1.5 5.0 10 15 20 25	Assumed
pp_k,i_	Percentage of population by activity class i for class k at start of HIV epidemic.	pp_k = 1, = 1...6 _= 55 20 10 10 2.5 2.5 pp_k = 2, = 1...6 _= 50 30 5 5 5 5	Assumed
*μ*	Rate of departure from sexually active population. Lifetime of sexual activity is 1/*μ *= 35 yrs.	*μ *= 0.02857	Assumed
Population	Size of population hosting clinical trial.	200 000	Assumed
Sample size	Number of individuals recruited to clinical trial (controls + circumcised).	2 750 with 1:1 randomization	[15,16]
Recruitment class	Trial participants are recruited from sex k = 2 and sexual activity classes i = 1,...,3 in same proportions as in pp_k,i_		
Follow-up	Maximum person-time contributed per individual.	2 years	[15,16]
Incidence- HIV	Average incidence rate of HIV in control group over course of clinical trial.	2.5%/year (This is a model output)	[15,16]
Incidence – STI	Average incidence rate of STI in control group over course of clinical trial.	22.0%/year (This is a model output)	
Prevalence – HIV	Prevalence of HIV in general population.	18% during course of trial (This is a model output)	[15,16,30]
Prevalence – STI	Prevalence of STI in control group	8.2 %. Under sensitivity analysis, prevalence is 1.8% and 19.0%. (This is a model output)	[15,16,30]
*E*_*H*_	Efficacy against HIV due to circumcision.	*E*_*H *_= 0%, 10%, 20%, ..., 70%	Assumed
*E*_*S*_	Efficacy against STI due to circumcision	*E*_*S *_= 0% to 80%	Assumed

The Kenyan circumcision study is an unblinded randomized controlled trial which began recruiting uncircumcised, HIV seronegative Luo men aged 18–24 years in February 2002. The Luo are a homogenous traditionally non-circumcising ethnic group in western Kenya. The primary objective is the determination of reduction in incidence of HIV infection, interpreted as effectiveness. Estimation of reduction in incidence of STI is a secondary objective. A total of 2784 participants are equally randomized to each arm of the trial and followed for 24 months. The estimated annual HIV infection rate in the control group is 2.5% yr^-1 ^and loss to follow-up is 15%. Given these trial parameters, 63 infection events would be expected in the control arm of the trial. This implies a 70% probability (power) of declaring an efficacy better than zero when the true effectiveness is 40%, or 90% power when true effectiveness is 50%. HIV prevalence in Luo men at screening was 7.4% and HIV incidence is estimated at 2.5%. The Kenya Demographic and Health Survey (DHS) [30] also reports HIV prevalence : Luo men aged 15–49 were reported to be 17.5% positive for HIV and uncircumcised Kenyan males aged 15–54 were 12.6% positive for HIV. STI prevalence in trial participants at enrolment was 2.0% urine positive for Neisseria gonorrhoea, 2.6% urine positive for Trachoma vaginalis (in pouch culture test), 2.8% rapid plasma reagent positive for syphilis, 5.0% urine positive for Chlamydia trachomatis and 25.0% antibody positive for HSV. The Kenya DHS also indicates 7.6% of males in Nyanza self-reported STI infection, abnormal genital discharge or ulcerative sores in the last year [30].

Model parameters for sample size, follow-up duration and randomisation ratio were chosen according to the trial's design characteristics. The size of the population hosting the trial was assumed to be much larger than the sample size of the study. The sexual behaviour parameters (i.e. distribution of the sexual activity classes and the rate of sexual partner acquisition) in the model were chosen to reproduce trial data and Kenyan DHS data on HIV prevalence, HIV incidence and STI prevalence. This parameter selection also ensured the simulated power, as determined by number of expected endpoints, were closely matched to the trial's actual power. Under base-case parameter values, the model's HIV prevalence in the general population averaged 18.0% over the time of the trial, HIV incidence rate in the simulated trials was 2.5% per year in control subjects, and STI prevalence in simulations averaged 8.2% in controls. Over the trial period HIV and STI prevalence remained unchanged. The simulated trials produced an average of 66 HIV infection events in the control arm.

Remaining parameters for HIV and STI transmission probabilities, duration of the different HIV states and duration of STI infection were estimated based on other published studies. In particular, the strength of interaction modelled between HIV and STI under base-case assumptions is assumed to be medium; presence of STI in the HIV-infected sexual partner increases the per partnership probability of HIV transmission by a factor of 4 (*α*_1 _= 4), while STI in the HIV-susceptible increases HIV transmission by a factor of 3 (*α*_1 _= 3). This compares with a review on the increased susceptibility to HIV due to STIs [13] where relative risk in men was 3.1 for all STIs, 4.4 for genital ulcer disease, 2.7 for herpes, 2.5 for syphilis, 3.9 for gonorrhoea and 0.8 for chlamydia. In our simulations, the STI had an average duration of infection of 6 months.

### Statistical analysis

We denote estimated values of *E*_*H*_, *E*_*S*_, *F *and *AF *by E^
 MathType@MTEF@5@5@+=feaafiart1ev1aaatCvAUfKttLearuWrP9MDH5MBPbIqV92AaeXatLxBI9gBaebbnrfifHhDYfgasaacH8akY=wiFfYdH8Gipec8Eeeu0xXdbba9frFj0=OqFfea0dXdd9vqai=hGuQ8kuc9pgc9s8qqaq=dirpe0xb9q8qiLsFr0=vr0=vr0dc8meaabaqaciaacaGaaeqabaqabeGadaaakeaacuWGfbqrgaqcaaaa@2DCF@_*H*_, E^
 MathType@MTEF@5@5@+=feaafiart1ev1aaatCvAUfKttLearuWrP9MDH5MBPbIqV92AaeXatLxBI9gBaebbnrfifHhDYfgasaacH8akY=wiFfYdH8Gipec8Eeeu0xXdbba9frFj0=OqFfea0dXdd9vqai=hGuQ8kuc9pgc9s8qqaq=dirpe0xb9q8qiLsFr0=vr0=vr0dc8meaabaqaciaacaGaaeqabaqabeGadaaakeaacuWGfbqrgaqcaaaa@2DCF@_*S*_, F^
 MathType@MTEF@5@5@+=feaafiart1ev1aaatCvAUfKttLearuWrP9MDH5MBPbIqV92AaeXatLxBI9gBaebbnrfifHhDYfgasaacH8akY=wiFfYdH8Gipec8Eeeu0xXdbba9frFj0=OqFfea0dXdd9vqai=hGuQ8kuc9pgc9s8qqaq=dirpe0xb9q8qiLsFr0=vr0=vr0dc8meaabaqaciaacaGaaeqabaqabeGadaaakeaacuWGgbGrgaqcaaaa@2DD1@ and AF^
 MathType@MTEF@5@5@+=feaafiart1ev1aaatCvAUfKttLearuWrP9MDH5MBPbIqV92AaeXatLxBI9gBaebbnrfifHhDYfgasaacH8akY=wiFfYdH8Gipec8Eeeu0xXdbba9frFj0=OqFfea0dXdd9vqai=hGuQ8kuc9pgc9s8qqaq=dirpe0xb9q8qiLsFr0=vr0=vr0dc8meaabaqaciaacaGaaeqabaqabeGadaaakeaadaqiaaqaaiabdgeabjabdAeagbGaayPadaaaaa@2F8E@ to distinguish them from their true values. Trial data generated by the model for each individual include the exact HIV infection time or right-censored times in case of loss to follow-up or trial closure, STI infection times and recovery times, and randomisation group. Estimation of *E*_*H *_is based on comparison of HIV infection times between trial arms where individuals' follow-up times are stratified into STI-positive and STI-negative intervals using exact STI infection and recovery times. Given the main endpoint is time to HIV infection where STI status is changing during the follow-up period and that multiple STI events are possible per individual, the Anderson-Gill counting process (a generalization of Cox proportional hazard models) with robust variance estimates is the most suitable method for estimation of *E*_*H *_[31,32]. The Anderson-Gill counting process is also the appropriate analysis for estimating *E*_*S*_. Estimation of *F *is based on comparison of HIV infection times between control and treated arms without stratification for STI infection. The proportion of HIV infections prevented which are attributable to MC efficacy against STI is approximated by AF^=F^−E^HF^
 MathType@MTEF@5@5@+=feaafiart1ev1aaatCvAUfKttLearuWrP9MDH5MBPbIqV92AaeXatLxBI9gBaebbnrfifHhDYfgasaacH8akY=wiFfYdH8Gipec8Eeeu0xXdbba9frFj0=OqFfea0dXdd9vqai=hGuQ8kuc9pgc9s8qqaq=dirpe0xb9q8qiLsFr0=vr0=vr0dc8meaabaqaciaacaGaaeqabaqabeGadaaakeaadaqiaaqaaiabdgeabjabdAeagbGaayPadaGaeyypa0ZaaSaaaeaacuWGgbGrgaqcaiabgkHiTiqbdweafzaajaWaaSbaaSqaaiabdIeaibqabaaakeaacuWGgbGrgaqcaaaaaaa@364D@. This approximation is valid under low incidence of HIV infection and low STI prevalence, as under the Kisumu base-case assumptions. Analyses were implemented with SAS version 8.1 [33].

### Simulations and sensitivity analysis

Values for *E*_*H*_, modelled as a reduction in susceptibility upon exposure varied between 0% and 70%. Similarly, values for *E*_*S *_between 0% to 80% were explored. Low *E*_*S *_(~0–20%) reflects the efficacy of MC on Chlamydia and HSV while high *E*_*S *_(~60–80%) reflects efficacy against chancroid and syphilis [8]. Forty scenarios of efficacy for *E*_*H *_and *E*_*S *_were simulated 100 times and analysed individually, giving 4000 simulated trials under base-case parameter values. The first objective was addressed by comparing the estimated E^
 MathType@MTEF@5@5@+=feaafiart1ev1aaatCvAUfKttLearuWrP9MDH5MBPbIqV92AaeXatLxBI9gBaebbnrfifHhDYfgasaacH8akY=wiFfYdH8Gipec8Eeeu0xXdbba9frFj0=OqFfea0dXdd9vqai=hGuQ8kuc9pgc9s8qqaq=dirpe0xb9q8qiLsFr0=vr0=vr0dc8meaabaqaciaacaGaaeqabaqabeGadaaakeaacuWGfbqrgaqcaaaa@2DCF@_*H *_against the true value for *E*_*H *_embedded in the 4,000 simulated trials. The second objective was addressed by examining the 5 scenarios where true *E*_*H *_= 0%. The third objective was addressed by the examining attributable fraction *AF *in the 4,000 simulated trials.

Further simulations to test the robustness of our conclusions were performed by univariate and bivariate sensitivity analyses (see Table 1). Under univariate analyses we varied parameters which influence STI prevalence and the role of STI in HIV transmission. Thus, we considered short (4 months) and long (1.3 years) duration of sexually transmitted infection resulting in STI prevalences of 1.8% and 19.0%, a higher strength of HIV-STI interaction (*α*_1 _= 6, *α*_2 _= 6) and a weaker HIV-STI interaction (*α*_1 _= 2, *α*_2 _= 2). Bivariate sensitivity analyses were additionally performed by varying the above parameters with compensating changes in HIV transmission probabilities in order to maintain annual HIV incidence in the trial constant at 2.5%, thus maintaining the power of the simulated study at a realistic level.

Additionally, for the first objective, the validity of efficacy estimation under 'inexact data' was assessed using interval-censored data assuming HIV and STI screening at 4, 6 and 8 months, corresponding to six, four and three testing times per 2 year follow-up. We also estimate efficacy controlling STI as a dichotomous variable representing 'STI ever' during follow-up.

## Results

### Validity of E^
 MathType@MTEF@5@5@+=feaafiart1ev1aaatCvAUfKttLearuWrP9MDH5MBPbIqV92AaeXatLxBI9gBaebbnrfifHhDYfgasaacH8akY=wiFfYdH8Gipec8Eeeu0xXdbba9frFj0=OqFfea0dXdd9vqai=hGuQ8kuc9pgc9s8qqaq=dirpe0xb9q8qiLsFr0=vr0=vr0dc8meaabaqaciaacaGaaeqabaqabeGadaaakeaacuWGfbqrgaqcaaaa@2DCF@_*H *_and E^
 MathType@MTEF@5@5@+=feaafiart1ev1aaatCvAUfKttLearuWrP9MDH5MBPbIqV92AaeXatLxBI9gBaebbnrfifHhDYfgasaacH8akY=wiFfYdH8Gipec8Eeeu0xXdbba9frFj0=OqFfea0dXdd9vqai=hGuQ8kuc9pgc9s8qqaq=dirpe0xb9q8qiLsFr0=vr0=vr0dc8meaabaqaciaacaGaaeqabaqabeGadaaakeaacuWGfbqrgaqcaaaa@2DCF@_*S *_estimates

In order to understand the relative importance of STIs in the prevention of HIV infections in phase 3 circumcision trials, we must be sure that the efficacy estimator of *E*_*H *_is statistically valid (i.e. unbiased). Table 2 gives the estimated E^
 MathType@MTEF@5@5@+=feaafiart1ev1aaatCvAUfKttLearuWrP9MDH5MBPbIqV92AaeXatLxBI9gBaebbnrfifHhDYfgasaacH8akY=wiFfYdH8Gipec8Eeeu0xXdbba9frFj0=OqFfea0dXdd9vqai=hGuQ8kuc9pgc9s8qqaq=dirpe0xb9q8qiLsFr0=vr0=vr0dc8meaabaqaciaacaGaaeqabaqabeGadaaakeaacuWGfbqrgaqcaaaa@2DCF@_*H *_where true *E*_*H *_varies from 0–70% and true *E*_*S *_varies from 0–80%. The estimates of *E*_*H *_reflect the true value embedded in each simulation, given that the median E^
 MathType@MTEF@5@5@+=feaafiart1ev1aaatCvAUfKttLearuWrP9MDH5MBPbIqV92AaeXatLxBI9gBaebbnrfifHhDYfgasaacH8akY=wiFfYdH8Gipec8Eeeu0xXdbba9frFj0=OqFfea0dXdd9vqai=hGuQ8kuc9pgc9s8qqaq=dirpe0xb9q8qiLsFr0=vr0=vr0dc8meaabaqaciaacaGaaeqabaqabeGadaaakeaacuWGfbqrgaqcaaaa@2DCF@_*H *_estimate over 100 simulations falls within ± 2.9% of the true value in all scenarios. Furthermore the E^
 MathType@MTEF@5@5@+=feaafiart1ev1aaatCvAUfKttLearuWrP9MDH5MBPbIqV92AaeXatLxBI9gBaebbnrfifHhDYfgasaacH8akY=wiFfYdH8Gipec8Eeeu0xXdbba9frFj0=OqFfea0dXdd9vqai=hGuQ8kuc9pgc9s8qqaq=dirpe0xb9q8qiLsFr0=vr0=vr0dc8meaabaqaciaacaGaaeqabaqabeGadaaakeaacuWGfbqrgaqcaaaa@2DCF@_*H *_estimates and their confidence limits were unchanged by efficacy against STI (reading down columns), contrary to the corresponding effectiveness estimates F^
 MathType@MTEF@5@5@+=feaafiart1ev1aaatCvAUfKttLearuWrP9MDH5MBPbIqV92AaeXatLxBI9gBaebbnrfifHhDYfgasaacH8akY=wiFfYdH8Gipec8Eeeu0xXdbba9frFj0=OqFfea0dXdd9vqai=hGuQ8kuc9pgc9s8qqaq=dirpe0xb9q8qiLsFr0=vr0=vr0dc8meaabaqaciaacaGaaeqabaqabeGadaaakeaacuWGgbGrgaqcaaaa@2DD1@ (Table 3). The effectiveness estimates generally overestimated efficacy by up to 13%, indicating the need to control for STI appropriately in statistical analyses.

**Table 2:** Estimates of efficacy against HIV (E^
 MathType@MTEF@5@5@+=feaafiart1ev1aaatCvAUfKttLearuWrP9MDH5MBPbIqV92AaeXatLxBI9gBaebbnrfifHhDYfgasaacH8akY=wiFfYdH8Gipec8Eeeu0xXdbba9frFj0=OqFfea0dXdd9vqai=hGuQ8kuc9pgc9s8qqaq=dirpe0xb9q8qiLsFr0=vr0=vr0dc8meaabaqaciaacaGaaeqabaqabeGadaaakeaacuWGfbqrgaqcaaaa@2DCF@_*H*_) and confidence intervals for simulated Kisumu MC trials under different levels of true efficacy against HIV (*E*_*H*_) and STI (*E*_*S*_) and base-case assumptions. Values for E^
 MathType@MTEF@5@5@+=feaafiart1ev1aaatCvAUfKttLearuWrP9MDH5MBPbIqV92AaeXatLxBI9gBaebbnrfifHhDYfgasaacH8akY=wiFfYdH8Gipec8Eeeu0xXdbba9frFj0=OqFfea0dXdd9vqai=hGuQ8kuc9pgc9s8qqaq=dirpe0xb9q8qiLsFr0=vr0=vr0dc8meaabaqaciaacaGaaeqabaqabeGadaaakeaacuWGfbqrgaqcaaaa@2DCF@_*H *_are the median values obtained over 100 repetitions while values in brackets are the median values for upper and lower limits of 95% confidence intervals for *E*_*H*_.

**Estimated **E^ MathType@MTEF@5@5@+=feaafiart1ev1aaatCvAUfKttLearuWrP9MDH5MBPbIqV92AaeXatLxBI9gBaebbnrfifHhDYfgasaacH8akY=wiFfYdH8Gipec8Eeeu0xXdbba9frFj0=OqFfea0dXdd9vqai=hGuQ8kuc9pgc9s8qqaq=dirpe0xb9q8qiLsFr0=vr0=vr0dc8meaabaqaciaacaGaaeqabaqabeGadaaakeaacuWGfbqrgaqcaaaa@2DCF@_*H *_**and 95% CI**		**Efficacy against HIV (true *E*_*H*_)**							
		
		**0%**	**10%**	**20%**	**30%**	**40%**	**50%**	**60%**	**70%**
**Efficacy against STI (true *E*_*S*_)**	**0%**	-1.4 (-43.2,27.6)	9.3 (-28.2,36.9)	21.0 (-13.5,43.7)	29.0 (-3.3,51.3)	37.6 (7.8,58.0)	49.6 (22.8,66.7)	58.5 (34.9,73.9)	69.6 (49.9,81.6)
	**20%**	-0.6 (-39.6,29.8)	9.9 (-27.6,35.9)	20.2 (-16.6,44.2)	32.2 (1.8,53.5)	39.1 (9.9,58.6)	51.6 (25.3,68.3)	59.8 (37.6,74.5)	70.7 (52.5,83.2)
	**40%**	0.5 (-45.1,29.5)	10.3 (-23.9,39.9)	19.9 (-16.1,44.6)	29.2 (-5.0,51.7)	37.3 (7.0,57.9)	48.0 (20.8,66.1)	59.0 (34.8,74.1)	68.8 (48.2,81.0)
	**60%**	-0.8 (-45.4,27.3)	8.6 (-30.6,36.4)	17.1 (-19.9,42.7)	30.9 (-2.2,54.4)	40.5 (10.1,60.6)	52.5 (25.0,68.7)	61.6 (39.6,76.3)	69.2 (48.0,81.6)
	**80%**	-0.4 (-46.4,26.8)	9.7 (-29.3,37.2)	18.1 (-19.8,44.1)	29.3 (-7.0,51.7)	40.5 (9.6,60.9)	48.4 (19.2,67.2)	58.7 (33.6,73.9)	69.5 (48.4,82.1)

**Table 3:** Estimates of effectiveness against HIV (F^
 MathType@MTEF@5@5@+=feaafiart1ev1aaatCvAUfKttLearuWrP9MDH5MBPbIqV92AaeXatLxBI9gBaebbnrfifHhDYfgasaacH8akY=wiFfYdH8Gipec8Eeeu0xXdbba9frFj0=OqFfea0dXdd9vqai=hGuQ8kuc9pgc9s8qqaq=dirpe0xb9q8qiLsFr0=vr0=vr0dc8meaabaqaciaacaGaaeqabaqabeGadaaakeaacuWGgbGrgaqcaaaa@2DD1@) and 95% confidence intervals for simulated Kisumu MC trials under different levels of true efficacy against HIV (*E*_*H*_) and STI (*E*_*S*_) and base-case assumptions. Values for F^
 MathType@MTEF@5@5@+=feaafiart1ev1aaatCvAUfKttLearuWrP9MDH5MBPbIqV92AaeXatLxBI9gBaebbnrfifHhDYfgasaacH8akY=wiFfYdH8Gipec8Eeeu0xXdbba9frFj0=OqFfea0dXdd9vqai=hGuQ8kuc9pgc9s8qqaq=dirpe0xb9q8qiLsFr0=vr0=vr0dc8meaabaqaciaacaGaaeqabaqabeGadaaakeaacuWGgbGrgaqcaaaa@2DD1@ are the median values obtained over 100 repetitions while values in brackets are the median values for upper and lower limits of 95% confidence intervals for *F*.

**Estimated **F^ MathType@MTEF@5@5@+=feaafiart1ev1aaatCvAUfKttLearuWrP9MDH5MBPbIqV92AaeXatLxBI9gBaebbnrfifHhDYfgasaacH8akY=wiFfYdH8Gipec8Eeeu0xXdbba9frFj0=OqFfea0dXdd9vqai=hGuQ8kuc9pgc9s8qqaq=dirpe0xb9q8qiLsFr0=vr0=vr0dc8meaabaqaciaacaGaaeqabaqabeGadaaakeaacuWGgbGrgaqcaaaa@2DD1@**and 95% CI**		**Efficacy against HIV (true *E*_*H*_)**							
		
		**0%**	**10%**	**20%**	**30%**	**40%**	**50%**	**60%**	**70%**
**Efficacy against STI (true *E*_*S*_)**	**0%**	-0.9 (-43.2,27.6)	8.8 (-28.2,35.8)	20.4 (-13.7,44.0)	28.6 (-4.2,51.2)	37.5 (7.4,58.2)	49.3 (22.7,66.7)	58.0 (34.6,73.6)	69.2 (48.5,81.2)
	**20%**	4.2 (-34.7,32.5)	12.3 (-25.3,38.1)	22.7 (-13.5,46.7)	34.8 (4.7,55.2)	40.6 (12.4,59.7)	52.8 (28.0,69.4)	61.0 (40.0,75.2)	71.7 (52.5,83.2)
	**40%**	5.6 (-34.4,34.3)	15.2 (-17.0,42.9)	25.4 (-6.7,49.3)	33.8 (3.3,54.8)	40.9 (12.5,60.3)	51.3 (26.0,68.6)	61.6 (39.3,76.0)	70.8 (52.2,82.2)
	**60%**	7.9 (-32.,35.3)	17.8 (-17.1,42.3)	27.1 (-5.1,49.8)	37.7 (7.2,57.7)	46.5 (20.1,64.1)	56.8 (33.2,71.6)	64.9 (44.9,78.3)	72.8 (53.5,83.4)
	**80%**	13.3 (-24.3,38.0)	22.4 (-11.6,45.2)	28.9 (-3.0,50.9)	38.7 (7.6,58.0)	49.4 (24.0,66.0)	56.3 (32.0,72.2)	64.7 (43.8,77.6)	73.9 (56.0,84.4)

However, when MC has no efficacy against STI (first row in Tables 2 &3), both E^
 MathType@MTEF@5@5@+=feaafiart1ev1aaatCvAUfKttLearuWrP9MDH5MBPbIqV92AaeXatLxBI9gBaebbnrfifHhDYfgasaacH8akY=wiFfYdH8Gipec8Eeeu0xXdbba9frFj0=OqFfea0dXdd9vqai=hGuQ8kuc9pgc9s8qqaq=dirpe0xb9q8qiLsFr0=vr0=vr0dc8meaabaqaciaacaGaaeqabaqabeGadaaakeaacuWGfbqrgaqcaaaa@2DCF@_*H *_and F^
 MathType@MTEF@5@5@+=feaafiart1ev1aaatCvAUfKttLearuWrP9MDH5MBPbIqV92AaeXatLxBI9gBaebbnrfifHhDYfgasaacH8akY=wiFfYdH8Gipec8Eeeu0xXdbba9frFj0=OqFfea0dXdd9vqai=hGuQ8kuc9pgc9s8qqaq=dirpe0xb9q8qiLsFr0=vr0=vr0dc8meaabaqaciaacaGaaeqabaqabeGadaaakeaacuWGgbGrgaqcaaaa@2DD1@ both validly reflect the true circumcision efficacy against HIV embedded in the simulation. The fact that E^
 MathType@MTEF@5@5@+=feaafiart1ev1aaatCvAUfKttLearuWrP9MDH5MBPbIqV92AaeXatLxBI9gBaebbnrfifHhDYfgasaacH8akY=wiFfYdH8Gipec8Eeeu0xXdbba9frFj0=OqFfea0dXdd9vqai=hGuQ8kuc9pgc9s8qqaq=dirpe0xb9q8qiLsFr0=vr0=vr0dc8meaabaqaciaacaGaaeqabaqabeGadaaakeaacuWGfbqrgaqcaaaa@2DCF@_*H *_and F^
 MathType@MTEF@5@5@+=feaafiart1ev1aaatCvAUfKttLearuWrP9MDH5MBPbIqV92AaeXatLxBI9gBaebbnrfifHhDYfgasaacH8akY=wiFfYdH8Gipec8Eeeu0xXdbba9frFj0=OqFfea0dXdd9vqai=hGuQ8kuc9pgc9s8qqaq=dirpe0xb9q8qiLsFr0=vr0=vr0dc8meaabaqaciaacaGaaeqabaqabeGadaaakeaacuWGgbGrgaqcaaaa@2DD1@ agree is evidence that the crude difference in HIV infection rates between trial arms is due only to the efficacy against HIV and there is no additional benefit in terms of protection against STI. This is confirmed by E^
 MathType@MTEF@5@5@+=feaafiart1ev1aaatCvAUfKttLearuWrP9MDH5MBPbIqV92AaeXatLxBI9gBaebbnrfifHhDYfgasaacH8akY=wiFfYdH8Gipec8Eeeu0xXdbba9frFj0=OqFfea0dXdd9vqai=hGuQ8kuc9pgc9s8qqaq=dirpe0xb9q8qiLsFr0=vr0=vr0dc8meaabaqaciaacaGaaeqabaqabeGadaaakeaacuWGfbqrgaqcaaaa@2DCF@_*S *_estimates (Table 4) indicating zero efficacy (with slight negative bias) against STI.

**Table 4:** Expected efficacy estimates against STI (E^
 MathType@MTEF@5@5@+=feaafiart1ev1aaatCvAUfKttLearuWrP9MDH5MBPbIqV92AaeXatLxBI9gBaebbnrfifHhDYfgasaacH8akY=wiFfYdH8Gipec8Eeeu0xXdbba9frFj0=OqFfea0dXdd9vqai=hGuQ8kuc9pgc9s8qqaq=dirpe0xb9q8qiLsFr0=vr0=vr0dc8meaabaqaciaacaGaaeqabaqabeGadaaakeaacuWGfbqrgaqcaaaa@2DCF@_*S*_) and 95% confidence intervals for simulated Kisumu MC trials under different levels of true efficacy against HIV (*E*_*H*_) and STI (*E*_*S*_) and base-case assumptions. Values for E^
 MathType@MTEF@5@5@+=feaafiart1ev1aaatCvAUfKttLearuWrP9MDH5MBPbIqV92AaeXatLxBI9gBaebbnrfifHhDYfgasaacH8akY=wiFfYdH8Gipec8Eeeu0xXdbba9frFj0=OqFfea0dXdd9vqai=hGuQ8kuc9pgc9s8qqaq=dirpe0xb9q8qiLsFr0=vr0=vr0dc8meaabaqaciaacaGaaeqabaqabeGadaaakeaacuWGfbqrgaqcaaaa@2DCF@_*S *_are the median values obtained over 100 repetitions while values in brackets are the median values for upper and lower limits of 95% confidence intervals for *E*_*S*_.

**Estimated **E^ MathType@MTEF@5@5@+=feaafiart1ev1aaatCvAUfKttLearuWrP9MDH5MBPbIqV92AaeXatLxBI9gBaebbnrfifHhDYfgasaacH8akY=wiFfYdH8Gipec8Eeeu0xXdbba9frFj0=OqFfea0dXdd9vqai=hGuQ8kuc9pgc9s8qqaq=dirpe0xb9q8qiLsFr0=vr0=vr0dc8meaabaqaciaacaGaaeqabaqabeGadaaakeaacuWGfbqrgaqcaaaa@2DCF@_*S *_**and 95% CI**		**Efficacy against HIV (true *E*_*H*_)**							
		
		**0%**	**10%**	**20%**	**30%**	**40%**	**50%**	**60%**	**70%**
**Efficacy against STI (true *E*_*S*_)**	**0%**	-0.5 (-19.1,15.2)	-0.8 (-19.7,14.8)	-1.2 (-18.6,15.1)	-0.2 (-18.6,15.1)	-2.0 (-20.4,14.2)	-1.7 (-20.1,13.9)	-2.0 (-20.1,13.9)	-4.7 (-23.8,11.4)
	**20%**	18.0 (2.1,31.7)	17.9 (1.5,31.2)	18.4 (2.6,31.8)	16.7 (0.6,30.5)	17.4 (1.1,30.6)	16.7 (0.6,30.6)	16.9 (0.1,30.5)	18.1 (1.9,31.4)
	**40%**	37.5 (24.2,48.5)	36.8 (23.3,47.9)	37.0 (23.5,47.9)	38.0 (24.4,52.0)	38.0 (24.6,48.9)	36.3 (22.8,47.6)	37.9 (24.8,48.9)	37.5 (42.2,48.4)
	**60%**	58.0 (47.9,66.3)	58.8 (48.8,66.9)	58.0 (47.8,66.2)	58.0 (47.6,66.2)	57.1 (46.7,65.5)	57.1 (46.7,65.5)	57.2 (46.7,65.5)	57.5 (47.0,65.5)
	**80%**	78.8 (71.9,84.2)	79.3 (72.4,84.4)	78.3 (71.1,83.6)	79.2 (72.2,84.1)	78.7 (71.8,84.0)	78.8 (71.6,84.0)	78.4 (71.4,83.5)	78.2 (71.1,83.6)

Table 4 gives E^
 MathType@MTEF@5@5@+=feaafiart1ev1aaatCvAUfKttLearuWrP9MDH5MBPbIqV92AaeXatLxBI9gBaebbnrfifHhDYfgasaacH8akY=wiFfYdH8Gipec8Eeeu0xXdbba9frFj0=OqFfea0dXdd9vqai=hGuQ8kuc9pgc9s8qqaq=dirpe0xb9q8qiLsFr0=vr0=vr0dc8meaabaqaciaacaGaaeqabaqabeGadaaakeaacuWGfbqrgaqcaaaa@2DCF@_*S *_estimates under different true *E*_*S *_and true *E*_*H*_. The true *E*_*S *_is slightly underestimated. This arises because of unbalanced censoring between trial arms. When analysing STI infection endpoints, censoring arises due to HIV infections which are linked to trial arm. In other words, when there is positive efficacy against HIV or an STI-cofactor, more censoring due to HIV infections will occur in the control arm compared to the circumcision arm, particularly reducing the person-time of higher risk uncircumcised men in the trial. Interestingly, the magnitude of the underestimation increases with *E*_*H*_, but never by more than 5%, under the conditions explored. Furthermore, given the greater incidence of STI infections compared to HIV, chances for detecting even small efficacies against STI, though negatively biased, will be good as reflected by the tight confidence intervals for *E*_*S *_in our simulated trials.

Under trial conditions, the exact STI and HIV infection times are unlikely to be available. Therefore we considered analyses based on data where HIV and STI infection times are interval-censored by 4, 6 or 8-month visit intervals. The efficacy estimates were typically between the corresponding values in Tables 2 and 3. Note however that the interval censored results frequently lie closer to the E^
 MathType@MTEF@5@5@+=feaafiart1ev1aaatCvAUfKttLearuWrP9MDH5MBPbIqV92AaeXatLxBI9gBaebbnrfifHhDYfgasaacH8akY=wiFfYdH8Gipec8Eeeu0xXdbba9frFj0=OqFfea0dXdd9vqai=hGuQ8kuc9pgc9s8qqaq=dirpe0xb9q8qiLsFr0=vr0=vr0dc8meaabaqaciaacaGaaeqabaqabeGadaaakeaacuWGfbqrgaqcaaaa@2DCF@_*H *_estimates based on exact time than to F^
 MathType@MTEF@5@5@+=feaafiart1ev1aaatCvAUfKttLearuWrP9MDH5MBPbIqV92AaeXatLxBI9gBaebbnrfifHhDYfgasaacH8akY=wiFfYdH8Gipec8Eeeu0xXdbba9frFj0=OqFfea0dXdd9vqai=hGuQ8kuc9pgc9s8qqaq=dirpe0xb9q8qiLsFr0=vr0=vr0dc8meaabaqaciaacaGaaeqabaqabeGadaaakeaacuWGgbGrgaqcaaaa@2DD1@ estimates. Since E^
 MathType@MTEF@5@5@+=feaafiart1ev1aaatCvAUfKttLearuWrP9MDH5MBPbIqV92AaeXatLxBI9gBaebbnrfifHhDYfgasaacH8akY=wiFfYdH8Gipec8Eeeu0xXdbba9frFj0=OqFfea0dXdd9vqai=hGuQ8kuc9pgc9s8qqaq=dirpe0xb9q8qiLsFr0=vr0=vr0dc8meaabaqaciaacaGaaeqabaqabeGadaaakeaacuWGfbqrgaqcaaaa@2DCF@_*H *_estimates made with interval censored data differed by ± 3% relative to exact time data, little statistical validity is in fact lost. Similarly, little validity was lost in estimation of *E*_*S *_using interval censored data compared to exact data. However, when STI control was attempted based on dichotomous variable representing ever having STI during follow-up, *E*_*H *_was consistently underestimated by ~5% compared to exact times. Thus even when data on HIV and STI infection times is interval censored, efficacy estimation is feasible, and STI infection times are preferable to 'STI-ever'. In other words, efficacy estimation using the Anderson-Gill counting process which computes each individual's HIV infection risk within STI-positive and STI-negative person-time intervals is preferable to a dichotomous STI indicator covariate. In sensitivity analyses, E^
 MathType@MTEF@5@5@+=feaafiart1ev1aaatCvAUfKttLearuWrP9MDH5MBPbIqV92AaeXatLxBI9gBaebbnrfifHhDYfgasaacH8akY=wiFfYdH8Gipec8Eeeu0xXdbba9frFj0=OqFfea0dXdd9vqai=hGuQ8kuc9pgc9s8qqaq=dirpe0xb9q8qiLsFr0=vr0=vr0dc8meaabaqaciaacaGaaeqabaqabeGadaaakeaacuWGfbqrgaqcaaaa@2DCF@_*H *_estimates remained within plus or minus 4% of the true *E*_*H*_, though estimates were more variable when incidence of HIV was lower in scenarios where STI prevalence was low or the strength of STI cofactor was weak.

### MC efficacy against STI only

When MC efficacy against HIV is zero (true *E*_*H *_= 0%, true *E*_*S *_> 0%; first column of Tables 2 &3), we observe that F^
 MathType@MTEF@5@5@+=feaafiart1ev1aaatCvAUfKttLearuWrP9MDH5MBPbIqV92AaeXatLxBI9gBaebbnrfifHhDYfgasaacH8akY=wiFfYdH8Gipec8Eeeu0xXdbba9frFj0=OqFfea0dXdd9vqai=hGuQ8kuc9pgc9s8qqaq=dirpe0xb9q8qiLsFr0=vr0=vr0dc8meaabaqaciaacaGaaeqabaqabeGadaaakeaacuWGgbGrgaqcaaaa@2DD1@ estimates diverge from the true efficacy value of zero as the efficacy of circumcision against STI (*E*_*S*_) increases. For example, when true *E*_*S *_= 40% the predicted trial effectiveness is around 6% (F^
 MathType@MTEF@5@5@+=feaafiart1ev1aaatCvAUfKttLearuWrP9MDH5MBPbIqV92AaeXatLxBI9gBaebbnrfifHhDYfgasaacH8akY=wiFfYdH8Gipec8Eeeu0xXdbba9frFj0=OqFfea0dXdd9vqai=hGuQ8kuc9pgc9s8qqaq=dirpe0xb9q8qiLsFr0=vr0=vr0dc8meaabaqaciaacaGaaeqabaqabeGadaaakeaacuWGgbGrgaqcaaaa@2DD1@ = 5.6%), while effectiveness is up to 13% (F^
 MathType@MTEF@5@5@+=feaafiart1ev1aaatCvAUfKttLearuWrP9MDH5MBPbIqV92AaeXatLxBI9gBaebbnrfifHhDYfgasaacH8akY=wiFfYdH8Gipec8Eeeu0xXdbba9frFj0=OqFfea0dXdd9vqai=hGuQ8kuc9pgc9s8qqaq=dirpe0xb9q8qiLsFr0=vr0=vr0dc8meaabaqaciaacaGaaeqabaqabeGadaaakeaacuWGgbGrgaqcaaaa@2DD1@ = 13.3%) when *E*_*S *_= 80%. Note however that for all true *E*_*S *_values, estimates of *E*_*H *_remain stable around zero (E^
 MathType@MTEF@5@5@+=feaafiart1ev1aaatCvAUfKttLearuWrP9MDH5MBPbIqV92AaeXatLxBI9gBaebbnrfifHhDYfgasaacH8akY=wiFfYdH8Gipec8Eeeu0xXdbba9frFj0=OqFfea0dXdd9vqai=hGuQ8kuc9pgc9s8qqaq=dirpe0xb9q8qiLsFr0=vr0=vr0dc8meaabaqaciaacaGaaeqabaqabeGadaaakeaacuWGfbqrgaqcaaaa@2DCF@_*H *_= -1.4 to 0.5%). In these simulations, the effectiveness is due entirely to the indirect protection against HIV achieved by MC efficacy against STI, but this can be ascertained only by observing the E^
 MathType@MTEF@5@5@+=feaafiart1ev1aaatCvAUfKttLearuWrP9MDH5MBPbIqV92AaeXatLxBI9gBaebbnrfifHhDYfgasaacH8akY=wiFfYdH8Gipec8Eeeu0xXdbba9frFj0=OqFfea0dXdd9vqai=hGuQ8kuc9pgc9s8qqaq=dirpe0xb9q8qiLsFr0=vr0=vr0dc8meaabaqaciaacaGaaeqabaqabeGadaaakeaacuWGfbqrgaqcaaaa@2DCF@_*H *_and E^
 MathType@MTEF@5@5@+=feaafiart1ev1aaatCvAUfKttLearuWrP9MDH5MBPbIqV92AaeXatLxBI9gBaebbnrfifHhDYfgasaacH8akY=wiFfYdH8Gipec8Eeeu0xXdbba9frFj0=OqFfea0dXdd9vqai=hGuQ8kuc9pgc9s8qqaq=dirpe0xb9q8qiLsFr0=vr0=vr0dc8meaabaqaciaacaGaaeqabaqabeGadaaakeaacuWGfbqrgaqcaaaa@2DCF@_*S *_estimates, which reflect their true values of *E*_*H *_and *E*_*S *_(e.g. F^
 MathType@MTEF@5@5@+=feaafiart1ev1aaatCvAUfKttLearuWrP9MDH5MBPbIqV92AaeXatLxBI9gBaebbnrfifHhDYfgasaacH8akY=wiFfYdH8Gipec8Eeeu0xXdbba9frFj0=OqFfea0dXdd9vqai=hGuQ8kuc9pgc9s8qqaq=dirpe0xb9q8qiLsFr0=vr0=vr0dc8meaabaqaciaacaGaaeqabaqabeGadaaakeaacuWGgbGrgaqcaaaa@2DD1@ = 13.3% when E^
 MathType@MTEF@5@5@+=feaafiart1ev1aaatCvAUfKttLearuWrP9MDH5MBPbIqV92AaeXatLxBI9gBaebbnrfifHhDYfgasaacH8akY=wiFfYdH8Gipec8Eeeu0xXdbba9frFj0=OqFfea0dXdd9vqai=hGuQ8kuc9pgc9s8qqaq=dirpe0xb9q8qiLsFr0=vr0=vr0dc8meaabaqaciaacaGaaeqabaqabeGadaaakeaacuWGfbqrgaqcaaaa@2DCF@_*H *_= -0.4% and E^
 MathType@MTEF@5@5@+=feaafiart1ev1aaatCvAUfKttLearuWrP9MDH5MBPbIqV92AaeXatLxBI9gBaebbnrfifHhDYfgasaacH8akY=wiFfYdH8Gipec8Eeeu0xXdbba9frFj0=OqFfea0dXdd9vqai=hGuQ8kuc9pgc9s8qqaq=dirpe0xb9q8qiLsFr0=vr0=vr0dc8meaabaqaciaacaGaaeqabaqabeGadaaakeaacuWGfbqrgaqcaaaa@2DCF@_*S *_= 78.8%). These results suggest that, under our parameter assumptions, even if MC has a very high efficacy against STI, it is unlikely to produce a large overall HIV effectiveness if circumcision does not also protect against HIV.

This seemingly small impact is understandable since the reduction in HIV infections during the two year follow-up period would, under these efficacy assumptions, be achieved only indirectly as the prevalence of STI in circumcised men declines relative to the controls. In our simulations, the STI prevalence at recruitment averaged 8.2% and remained constant in control subjects. However, in circumcised men, STI prevalence declined over two years to 2.9% when true E^
 MathType@MTEF@5@5@+=feaafiart1ev1aaatCvAUfKttLearuWrP9MDH5MBPbIqV92AaeXatLxBI9gBaebbnrfifHhDYfgasaacH8akY=wiFfYdH8Gipec8Eeeu0xXdbba9frFj0=OqFfea0dXdd9vqai=hGuQ8kuc9pgc9s8qqaq=dirpe0xb9q8qiLsFr0=vr0=vr0dc8meaabaqaciaacaGaaeqabaqabeGadaaakeaacuWGfbqrgaqcaaaa@2DCF@_*S *_= 80%, as new STI infections were substantially reduced and as prevalent infections recovered with an average duration of six months.

To further explore why STIs play a small role in preventing HIV infection, we approximate the fraction of all HIV infections attributable to the STI in the trial. We do this by 'removing' the HIV-STI interaction effect in circumcised men by simulating perfect efficacy against STI and zero efficacy against HIV (*E*_*S *_= 100%, *E*_*H *_= 0%) and increasing the follow-up period to five years. This resulted in STI prevalence of 0.7% at five years in circumcised men and an estimated F^
 MathType@MTEF@5@5@+=feaafiart1ev1aaatCvAUfKttLearuWrP9MDH5MBPbIqV92AaeXatLxBI9gBaebbnrfifHhDYfgasaacH8akY=wiFfYdH8Gipec8Eeeu0xXdbba9frFj0=OqFfea0dXdd9vqai=hGuQ8kuc9pgc9s8qqaq=dirpe0xb9q8qiLsFr0=vr0=vr0dc8meaabaqaciaacaGaaeqabaqabeGadaaakeaacuWGgbGrgaqcaaaa@2DD1@ = 21.0%. We interpret this figure both as the proportion of all HIV infections attributable to STI coinfection and as the upper limit of effectiveness which would be possible in a MC trial where MC has no protective efficacy against HIV. Our sensitivity analyses demonstrated that this upper limit of effectiveness could be as high as 25% to 30% under high strength of HIV-STI interaction effect or high STI prevalence, but these scenarios were associated with over 10% annual HIV incidence and would therefore not appear to be realistic for current MC trials.

### Attributable fraction

When both efficacy mechanisms combine (true *E*_*H *_> 0%, true *E*_*S *_> 0%), the interpretation of effectiveness becomes more complex. Different combinations of true *E*_*H *_and true *E*_*S *_can produce similar F^
 MathType@MTEF@5@5@+=feaafiart1ev1aaatCvAUfKttLearuWrP9MDH5MBPbIqV92AaeXatLxBI9gBaebbnrfifHhDYfgasaacH8akY=wiFfYdH8Gipec8Eeeu0xXdbba9frFj0=OqFfea0dXdd9vqai=hGuQ8kuc9pgc9s8qqaq=dirpe0xb9q8qiLsFr0=vr0=vr0dc8meaabaqaciaacaGaaeqabaqabeGadaaakeaacuWGgbGrgaqcaaaa@2DD1@ estimates. For example, an estimated overall effectiveness F^
 MathType@MTEF@5@5@+=feaafiart1ev1aaatCvAUfKttLearuWrP9MDH5MBPbIqV92AaeXatLxBI9gBaebbnrfifHhDYfgasaacH8akY=wiFfYdH8Gipec8Eeeu0xXdbba9frFj0=OqFfea0dXdd9vqai=hGuQ8kuc9pgc9s8qqaq=dirpe0xb9q8qiLsFr0=vr0=vr0dc8meaabaqaciaacaGaaeqabaqabeGadaaakeaacuWGgbGrgaqcaaaa@2DD1@ around 40% can be the result of either true *E*_*H *_= 40% and *E*_*S *_= 0–20% or *E*_*H *_= 30% and *E*_*S *_= 60–80% (Tables 2 &3). In general, several combinations of efficacies will give rise to a given effectiveness, particularly where the intervention is neither strongly nor weakly effective.

We also note that the greater the true *E*_*S *_and weaker the true *E*_*H *_then the greater is the difference between F^
 MathType@MTEF@5@5@+=feaafiart1ev1aaatCvAUfKttLearuWrP9MDH5MBPbIqV92AaeXatLxBI9gBaebbnrfifHhDYfgasaacH8akY=wiFfYdH8Gipec8Eeeu0xXdbba9frFj0=OqFfea0dXdd9vqai=hGuQ8kuc9pgc9s8qqaq=dirpe0xb9q8qiLsFr0=vr0=vr0dc8meaabaqaciaacaGaaeqabaqabeGadaaakeaacuWGgbGrgaqcaaaa@2DD1@ estimates and true *E*_*H*_. For example, estimates of F^
 MathType@MTEF@5@5@+=feaafiart1ev1aaatCvAUfKttLearuWrP9MDH5MBPbIqV92AaeXatLxBI9gBaebbnrfifHhDYfgasaacH8akY=wiFfYdH8Gipec8Eeeu0xXdbba9frFj0=OqFfea0dXdd9vqai=hGuQ8kuc9pgc9s8qqaq=dirpe0xb9q8qiLsFr0=vr0=vr0dc8meaabaqaciaacaGaaeqabaqabeGadaaakeaacuWGgbGrgaqcaaaa@2DD1@ are around 22.4% when true *E*_*H *_= 10% and true *E*_*S *_= 80% compared to F^
 MathType@MTEF@5@5@+=feaafiart1ev1aaatCvAUfKttLearuWrP9MDH5MBPbIqV92AaeXatLxBI9gBaebbnrfifHhDYfgasaacH8akY=wiFfYdH8Gipec8Eeeu0xXdbba9frFj0=OqFfea0dXdd9vqai=hGuQ8kuc9pgc9s8qqaq=dirpe0xb9q8qiLsFr0=vr0=vr0dc8meaabaqaciaacaGaaeqabaqabeGadaaakeaacuWGgbGrgaqcaaaa@2DD1@ = 8.8% if true *E*_*H *_= 10% and *E*_*S *_= 0%. In both cases, E^
 MathType@MTEF@5@5@+=feaafiart1ev1aaatCvAUfKttLearuWrP9MDH5MBPbIqV92AaeXatLxBI9gBaebbnrfifHhDYfgasaacH8akY=wiFfYdH8Gipec8Eeeu0xXdbba9frFj0=OqFfea0dXdd9vqai=hGuQ8kuc9pgc9s8qqaq=dirpe0xb9q8qiLsFr0=vr0=vr0dc8meaabaqaciaacaGaaeqabaqabeGadaaakeaacuWGfbqrgaqcaaaa@2DCF@_*H *_reflected true *E*_*H *_(E^
 MathType@MTEF@5@5@+=feaafiart1ev1aaatCvAUfKttLearuWrP9MDH5MBPbIqV92AaeXatLxBI9gBaebbnrfifHhDYfgasaacH8akY=wiFfYdH8Gipec8Eeeu0xXdbba9frFj0=OqFfea0dXdd9vqai=hGuQ8kuc9pgc9s8qqaq=dirpe0xb9q8qiLsFr0=vr0=vr0dc8meaabaqaciaacaGaaeqabaqabeGadaaakeaacuWGfbqrgaqcaaaa@2DCF@_*H *_= 9.3% and 9.7%) while E^
 MathType@MTEF@5@5@+=feaafiart1ev1aaatCvAUfKttLearuWrP9MDH5MBPbIqV92AaeXatLxBI9gBaebbnrfifHhDYfgasaacH8akY=wiFfYdH8Gipec8Eeeu0xXdbba9frFj0=OqFfea0dXdd9vqai=hGuQ8kuc9pgc9s8qqaq=dirpe0xb9q8qiLsFr0=vr0=vr0dc8meaabaqaciaacaGaaeqabaqabeGadaaakeaacuWGfbqrgaqcaaaa@2DCF@_*S *_reflected true *E*_*S *_(E^
 MathType@MTEF@5@5@+=feaafiart1ev1aaatCvAUfKttLearuWrP9MDH5MBPbIqV92AaeXatLxBI9gBaebbnrfifHhDYfgasaacH8akY=wiFfYdH8Gipec8Eeeu0xXdbba9frFj0=OqFfea0dXdd9vqai=hGuQ8kuc9pgc9s8qqaq=dirpe0xb9q8qiLsFr0=vr0=vr0dc8meaabaqaciaacaGaaeqabaqabeGadaaakeaacuWGfbqrgaqcaaaa@2DCF@_*S *_= -0.8 and 79.3%). The validity of the efficacy estimates make apparent when the majority of HIV infections averted in the circumcised arm of the trial were due to protection against STI, and this can be quantified in the *AF*.

**Figure 1:**
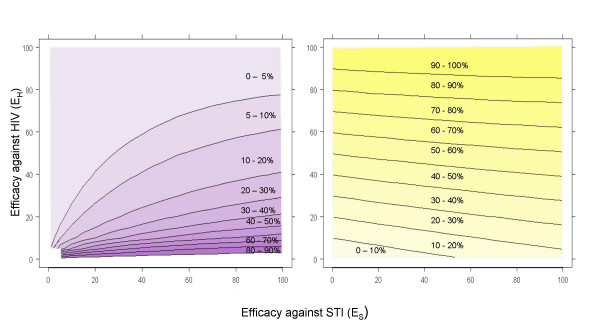
Attributable Fraction and Effectiveness Planes. Left Panel is attributable fraction plane showing regions of low (ligher purple) to high (darker purple) percentage of HIV infections prevented which are attributable in to MC efficacy against STI at different combinations of MC efficacy against HIV (*E*_*H*_) and STI (*E*_*S*_). Right panel is plane showing regions of low (lighter gold) to high (darker gold) effectiveness at different combinations of MC efficacy against HIV (*E*_*H*_) and STI (*E*_*S*_).

Figure 1 plots several values for AF^
 MathType@MTEF@5@5@+=feaafiart1ev1aaatCvAUfKttLearuWrP9MDH5MBPbIqV92AaeXatLxBI9gBaebbnrfifHhDYfgasaacH8akY=wiFfYdH8Gipec8Eeeu0xXdbba9frFj0=OqFfea0dXdd9vqai=hGuQ8kuc9pgc9s8qqaq=dirpe0xb9q8qiLsFr0=vr0=vr0dc8meaabaqaciaacaGaaeqabaqabeGadaaakeaadaqiaaqaaiabdgeabjabdAeagbGaayPadaaaaa@2F8E@ (calculated from the estimated E^
 MathType@MTEF@5@5@+=feaafiart1ev1aaatCvAUfKttLearuWrP9MDH5MBPbIqV92AaeXatLxBI9gBaebbnrfifHhDYfgasaacH8akY=wiFfYdH8Gipec8Eeeu0xXdbba9frFj0=OqFfea0dXdd9vqai=hGuQ8kuc9pgc9s8qqaq=dirpe0xb9q8qiLsFr0=vr0=vr0dc8meaabaqaciaacaGaaeqabaqabeGadaaakeaacuWGfbqrgaqcaaaa@2DCF@_*H *_and F^
 MathType@MTEF@5@5@+=feaafiart1ev1aaatCvAUfKttLearuWrP9MDH5MBPbIqV92AaeXatLxBI9gBaebbnrfifHhDYfgasaacH8akY=wiFfYdH8Gipec8Eeeu0xXdbba9frFj0=OqFfea0dXdd9vqai=hGuQ8kuc9pgc9s8qqaq=dirpe0xb9q8qiLsFr0=vr0=vr0dc8meaabaqaciaacaGaaeqabaqabeGadaaakeaacuWGgbGrgaqcaaaa@2DD1@), illustrating regions of equivalent proportions of HIV infections prevented due to MC efficacy against STI, as a function of true *E*_*H *_and *E*_*S *_and under base-case parameter assumptions. The lightest purple region indicates when AF^
 MathType@MTEF@5@5@+=feaafiart1ev1aaatCvAUfKttLearuWrP9MDH5MBPbIqV92AaeXatLxBI9gBaebbnrfifHhDYfgasaacH8akY=wiFfYdH8Gipec8Eeeu0xXdbba9frFj0=OqFfea0dXdd9vqai=hGuQ8kuc9pgc9s8qqaq=dirpe0xb9q8qiLsFr0=vr0=vr0dc8meaabaqaciaacaGaaeqabaqabeGadaaakeaadaqiaaqaaiabdgeabjabdAeagbGaayPadaaaaa@2F8E@ will be 0–5%, while the darkest shows when AF^
 MathType@MTEF@5@5@+=feaafiart1ev1aaatCvAUfKttLearuWrP9MDH5MBPbIqV92AaeXatLxBI9gBaebbnrfifHhDYfgasaacH8akY=wiFfYdH8Gipec8Eeeu0xXdbba9frFj0=OqFfea0dXdd9vqai=hGuQ8kuc9pgc9s8qqaq=dirpe0xb9q8qiLsFr0=vr0=vr0dc8meaabaqaciaacaGaaeqabaqabeGadaaakeaadaqiaaqaaiabdgeabjabdAeagbGaayPadaaaaa@2F8E@ will be 80–90%. In general, *E*_*H *_must be below 20% for a substantial proportion of HIV infections to be prevented via STI. For example, if efficacy against HIV and STI are 10% and 80%, respectively, then about 60% of HIV infections would be prevented in the circumcised arm due to MC efficacy against STI. The AF^
 MathType@MTEF@5@5@+=feaafiart1ev1aaatCvAUfKttLearuWrP9MDH5MBPbIqV92AaeXatLxBI9gBaebbnrfifHhDYfgasaacH8akY=wiFfYdH8Gipec8Eeeu0xXdbba9frFj0=OqFfea0dXdd9vqai=hGuQ8kuc9pgc9s8qqaq=dirpe0xb9q8qiLsFr0=vr0=vr0dc8meaabaqaciaacaGaaeqabaqabeGadaaakeaadaqiaaqaaiabdgeabjabdAeagbGaayPadaaaaa@2F8E@ declines very quickly as *E*_*H *_increases. If *E*_*H *_is above 40%, then at most 21% of HIV cases prevented in the circumcised arm could be expected to be due to STI, even if true *E*_*S *_is as high as 100%. Figure 1 also shows the regions of equivalent effectiveness resulting from different combinations of *E*_*H *_and *E*_*S *_in a two year MC trial. The lightest gold region shows when total effectiveness will be 0–10%, while the darkest indicates when F^
 MathType@MTEF@5@5@+=feaafiart1ev1aaatCvAUfKttLearuWrP9MDH5MBPbIqV92AaeXatLxBI9gBaebbnrfifHhDYfgasaacH8akY=wiFfYdH8Gipec8Eeeu0xXdbba9frFj0=OqFfea0dXdd9vqai=hGuQ8kuc9pgc9s8qqaq=dirpe0xb9q8qiLsFr0=vr0=vr0dc8meaabaqaciaacaGaaeqabaqabeGadaaakeaacuWGgbGrgaqcaaaa@2DD1@ will be 90–100%. The regions are almost horizontal, particularly at high efficacy against HIV, suggesting that efficacy against STI will play a minor role in overall effectiveness. Both panels of Figure 1 together illustrate that even in regions where AF^
 MathType@MTEF@5@5@+=feaafiart1ev1aaatCvAUfKttLearuWrP9MDH5MBPbIqV92AaeXatLxBI9gBaebbnrfifHhDYfgasaacH8akY=wiFfYdH8Gipec8Eeeu0xXdbba9frFj0=OqFfea0dXdd9vqai=hGuQ8kuc9pgc9s8qqaq=dirpe0xb9q8qiLsFr0=vr0=vr0dc8meaabaqaciaacaGaaeqabaqabeGadaaakeaadaqiaaqaaiabdgeabjabdAeagbGaayPadaaaaa@2F8E@ are high, the overall effectiveness remains low. For example, when *E*_*H *_= 15%, AF^
 MathType@MTEF@5@5@+=feaafiart1ev1aaatCvAUfKttLearuWrP9MDH5MBPbIqV92AaeXatLxBI9gBaebbnrfifHhDYfgasaacH8akY=wiFfYdH8Gipec8Eeeu0xXdbba9frFj0=OqFfea0dXdd9vqai=hGuQ8kuc9pgc9s8qqaq=dirpe0xb9q8qiLsFr0=vr0=vr0dc8meaabaqaciaacaGaaeqabaqabeGadaaakeaadaqiaaqaaiabdgeabjabdAeagbGaayPadaaaaa@2F8E@ may be as high as 60–70%, but F^
 MathType@MTEF@5@5@+=feaafiart1ev1aaatCvAUfKttLearuWrP9MDH5MBPbIqV92AaeXatLxBI9gBaebbnrfifHhDYfgasaacH8akY=wiFfYdH8Gipec8Eeeu0xXdbba9frFj0=OqFfea0dXdd9vqai=hGuQ8kuc9pgc9s8qqaq=dirpe0xb9q8qiLsFr0=vr0=vr0dc8meaabaqaciaacaGaaeqabaqabeGadaaakeaacuWGgbGrgaqcaaaa@2DD1@ would be unlikely be greater than 20–30%. Furthermore, it is only in regions where AF^
 MathType@MTEF@5@5@+=feaafiart1ev1aaatCvAUfKttLearuWrP9MDH5MBPbIqV92AaeXatLxBI9gBaebbnrfifHhDYfgasaacH8akY=wiFfYdH8Gipec8Eeeu0xXdbba9frFj0=OqFfea0dXdd9vqai=hGuQ8kuc9pgc9s8qqaq=dirpe0xb9q8qiLsFr0=vr0=vr0dc8meaabaqaciaacaGaaeqabaqabeGadaaakeaadaqiaaqaaiabdgeabjabdAeagbGaayPadaaaaa@2F8E@ is low that high efficacy is possible for example, if true *E*_*H *_= 60% and true *E*_*S *_= 60%, then F^
 MathType@MTEF@5@5@+=feaafiart1ev1aaatCvAUfKttLearuWrP9MDH5MBPbIqV92AaeXatLxBI9gBaebbnrfifHhDYfgasaacH8akY=wiFfYdH8Gipec8Eeeu0xXdbba9frFj0=OqFfea0dXdd9vqai=hGuQ8kuc9pgc9s8qqaq=dirpe0xb9q8qiLsFr0=vr0=vr0dc8meaabaqaciaacaGaaeqabaqabeGadaaakeaacuWGgbGrgaqcaaaa@2DD1@ is 5–10% while AF^
 MathType@MTEF@5@5@+=feaafiart1ev1aaatCvAUfKttLearuWrP9MDH5MBPbIqV92AaeXatLxBI9gBaebbnrfifHhDYfgasaacH8akY=wiFfYdH8Gipec8Eeeu0xXdbba9frFj0=OqFfea0dXdd9vqai=hGuQ8kuc9pgc9s8qqaq=dirpe0xb9q8qiLsFr0=vr0=vr0dc8meaabaqaciaacaGaaeqabaqabeGadaaakeaadaqiaaqaaiabdgeabjabdAeagbGaayPadaaaaa@2F8E@ is 60–70%. Thus, under our base-case parameter assumptions, a large observed effectiveness is unlikely to be possible without a significant MC efficacy against HIV, whatever the MC efficacy against STI.

Figure 1 also shows that a 60% effectiveness (c.f. Orange Farm MC trial reported 61% effectiveness) could have been the result of 60% efficacy against HIV and no efficacy against STI, at one extreme, and 50% efficacy against HIV combined with nearly 100% efficacy against STI, at the other. Similarly, an effectiveness of 50% (c.f. Kisumu and Rakai MC trials found 53% and 48% effectiveness) could be the result of 50% efficacy against HIV and no efficacy against STI or 39% efficacy against HIV and 100% efficacy against STI. The corresponding AF^
 MathType@MTEF@5@5@+=feaafiart1ev1aaatCvAUfKttLearuWrP9MDH5MBPbIqV92AaeXatLxBI9gBaebbnrfifHhDYfgasaacH8akY=wiFfYdH8Gipec8Eeeu0xXdbba9frFj0=OqFfea0dXdd9vqai=hGuQ8kuc9pgc9s8qqaq=dirpe0xb9q8qiLsFr0=vr0=vr0dc8meaabaqaciaacaGaaeqabaqabeGadaaakeaadaqiaaqaaiabdgeabjabdAeagbGaayPadaaaaa@2F8E@ in such cases could not be more than 10–20%. Thus, assuming similarity of Kisumu, Orange Farm and Rakai MC trials with respect to their design and epidemiological conditions, STI probably played a minor role in the significant effectiveness estimates from the three landmark MC studies.

Simulations performed for sensitivity analyses showed that conditions which increase STI prevalence or the strength of STI cofactor effect increase, predictably, the *AF*, but this change was only moderate. For example, when true *E*_*H *_= 50% and *E*_*S *_= 80%, the AF^
 MathType@MTEF@5@5@+=feaafiart1ev1aaatCvAUfKttLearuWrP9MDH5MBPbIqV92AaeXatLxBI9gBaebbnrfifHhDYfgasaacH8akY=wiFfYdH8Gipec8Eeeu0xXdbba9frFj0=OqFfea0dXdd9vqai=hGuQ8kuc9pgc9s8qqaq=dirpe0xb9q8qiLsFr0=vr0=vr0dc8meaabaqaciaacaGaaeqabaqabeGadaaakeaadaqiaaqaaiabdgeabjabdAeagbGaayPadaaaaa@2F8E@ was 11.7% under base-case assumptions, but AF^
 MathType@MTEF@5@5@+=feaafiart1ev1aaatCvAUfKttLearuWrP9MDH5MBPbIqV92AaeXatLxBI9gBaebbnrfifHhDYfgasaacH8akY=wiFfYdH8Gipec8Eeeu0xXdbba9frFj0=OqFfea0dXdd9vqai=hGuQ8kuc9pgc9s8qqaq=dirpe0xb9q8qiLsFr0=vr0=vr0dc8meaabaqaciaacaGaaeqabaqabeGadaaakeaadaqiaaqaaiabdgeabjabdAeagbGaayPadaaaaa@2F8E@ was 13.0% to 16.9% under high STI prevalence or high strength of HIV-STI interaction effect. Generally, the AF^
 MathType@MTEF@5@5@+=feaafiart1ev1aaatCvAUfKttLearuWrP9MDH5MBPbIqV92AaeXatLxBI9gBaebbnrfifHhDYfgasaacH8akY=wiFfYdH8Gipec8Eeeu0xXdbba9frFj0=OqFfea0dXdd9vqai=hGuQ8kuc9pgc9s8qqaq=dirpe0xb9q8qiLsFr0=vr0=vr0dc8meaabaqaciaacaGaaeqabaqabeGadaaakeaadaqiaaqaaiabdgeabjabdAeagbGaayPadaaaaa@2F8E@ increased by only 3% to 11% in scenarios with the heightened role of STI compared to the base-case assumptions.

Under conditions which lessen the importance of STI (i.e. lower STI prevalence and weaker HIV-STI interaction effect), the AF^
 MathType@MTEF@5@5@+=feaafiart1ev1aaatCvAUfKttLearuWrP9MDH5MBPbIqV92AaeXatLxBI9gBaebbnrfifHhDYfgasaacH8akY=wiFfYdH8Gipec8Eeeu0xXdbba9frFj0=OqFfea0dXdd9vqai=hGuQ8kuc9pgc9s8qqaq=dirpe0xb9q8qiLsFr0=vr0=vr0dc8meaabaqaciaacaGaaeqabaqabeGadaaakeaadaqiaaqaaiabdgeabjabdAeagbGaayPadaaaaa@2F8E@ varied from 3.2% to 6.6% under the same efficacy assumptions. It was generally the case in univariate and bivariate sensitivity analyses that AF^
 MathType@MTEF@5@5@+=feaafiart1ev1aaatCvAUfKttLearuWrP9MDH5MBPbIqV92AaeXatLxBI9gBaebbnrfifHhDYfgasaacH8akY=wiFfYdH8Gipec8Eeeu0xXdbba9frFj0=OqFfea0dXdd9vqai=hGuQ8kuc9pgc9s8qqaq=dirpe0xb9q8qiLsFr0=vr0=vr0dc8meaabaqaciaacaGaaeqabaqabeGadaaakeaadaqiaaqaaiabdgeabjabdAeagbGaayPadaaaaa@2F8E@ was not much affected by changes in parameter assumptions, except when true *E*_*H *_was very small (between 0 to 20%). Under scenarios of lessened importance of STI, F^
 MathType@MTEF@5@5@+=feaafiart1ev1aaatCvAUfKttLearuWrP9MDH5MBPbIqV92AaeXatLxBI9gBaebbnrfifHhDYfgasaacH8akY=wiFfYdH8Gipec8Eeeu0xXdbba9frFj0=OqFfea0dXdd9vqai=hGuQ8kuc9pgc9s8qqaq=dirpe0xb9q8qiLsFr0=vr0=vr0dc8meaabaqaciaacaGaaeqabaqabeGadaaakeaacuWGgbGrgaqcaaaa@2DD1@ and E^
 MathType@MTEF@5@5@+=feaafiart1ev1aaatCvAUfKttLearuWrP9MDH5MBPbIqV92AaeXatLxBI9gBaebbnrfifHhDYfgasaacH8akY=wiFfYdH8Gipec8Eeeu0xXdbba9frFj0=OqFfea0dXdd9vqai=hGuQ8kuc9pgc9s8qqaq=dirpe0xb9q8qiLsFr0=vr0=vr0dc8meaabaqaciaacaGaaeqabaqabeGadaaakeaacuWGfbqrgaqcaaaa@2DCF@_*H *_estimates generally did not differ by more than 3%. Closeness of E^
 MathType@MTEF@5@5@+=feaafiart1ev1aaatCvAUfKttLearuWrP9MDH5MBPbIqV92AaeXatLxBI9gBaebbnrfifHhDYfgasaacH8akY=wiFfYdH8Gipec8Eeeu0xXdbba9frFj0=OqFfea0dXdd9vqai=hGuQ8kuc9pgc9s8qqaq=dirpe0xb9q8qiLsFr0=vr0=vr0dc8meaabaqaciaacaGaaeqabaqabeGadaaakeaacuWGfbqrgaqcaaaa@2DCF@_*H *_and F^
 MathType@MTEF@5@5@+=feaafiart1ev1aaatCvAUfKttLearuWrP9MDH5MBPbIqV92AaeXatLxBI9gBaebbnrfifHhDYfgasaacH8akY=wiFfYdH8Gipec8Eeeu0xXdbba9frFj0=OqFfea0dXdd9vqai=hGuQ8kuc9pgc9s8qqaq=dirpe0xb9q8qiLsFr0=vr0=vr0dc8meaabaqaciaacaGaaeqabaqabeGadaaakeaacuWGgbGrgaqcaaaa@2DD1@ in these scenarios did not diminish the relevance of efficacy estimation for both STI and HIV since the efficacy estimates served to explain whether most of the effectiveness was due to direct reduction of susceptibility against HIV and whether the similarity between effectiveness and efficacy is due to low *E*_*S *_or to weak STI-HIV interaction.

## Conclusion

Our findings have implications first for the interpretation and analysis of circumcision trials and second for understanding the broader public health impact of MC. In the context of the current male circumcision trials and under our epidemiological assumptions, our results suggest that the protective efficacy of MC against STI alone is unlikely to produce large overall estimates of HIV effectiveness. For instance, effectiveness would be estimated at less than ~20%, even if the protective effect of MC against STI is as high as 80%. Moreover, the fraction of HIV infections attributable to MC efficacy against STI is unlikely to be high, except when protective efficacy of MC against HIV is small (*E*_*H *_~ 0–20%). A corollary is if a MC trial demonstrates a moderate to high effectiveness (F^
 MathType@MTEF@5@5@+=feaafiart1ev1aaatCvAUfKttLearuWrP9MDH5MBPbIqV92AaeXatLxBI9gBaebbnrfifHhDYfgasaacH8akY=wiFfYdH8Gipec8Eeeu0xXdbba9frFj0=OqFfea0dXdd9vqai=hGuQ8kuc9pgc9s8qqaq=dirpe0xb9q8qiLsFr0=vr0=vr0dc8meaabaqaciaacaGaaeqabaqabeGadaaakeaacuWGgbGrgaqcaaaa@2DD1@ ~ 40% or more), then only a minority of HIV infections in the trial are likely to have been prevented by protection against STI. In other words, a moderate to high effectiveness can be achieved only with moderate to high MC efficacy against HIV. Thus, assuming similarity between Kisumu and Orange Farm studies in terms of HIV and STI epidemiology, the 61% effectiveness of MC found in the Orange Farm study was mostly due to an efficacy against HIV of 50–60%. Similarly, the 53% and 48% effectiveness found in the Kisumu and Rakai studies were mostly due to efficacy against HIV of 39% to 53%. Our sensitivity analysis shows this is likely to be the case even with higher STI cofactor effect and higher STI prevalence.

These conclusions on the importance of STI for HIV effectiveness could be made only because estimation of the separate efficacies of MC against HIV and STI was possible. Our results also help guide the choice of statistical analysis and their interpretation. Estimation of *F *alone may lead to equivocal interpretations as we may not know how much an observed effectiveness was due to direct protection against HIV or indirect protection via STI reduction. Furthermore, the F^
 MathType@MTEF@5@5@+=feaafiart1ev1aaatCvAUfKttLearuWrP9MDH5MBPbIqV92AaeXatLxBI9gBaebbnrfifHhDYfgasaacH8akY=wiFfYdH8Gipec8Eeeu0xXdbba9frFj0=OqFfea0dXdd9vqai=hGuQ8kuc9pgc9s8qqaq=dirpe0xb9q8qiLsFr0=vr0=vr0dc8meaabaqaciaacaGaaeqabaqabeGadaaakeaacuWGgbGrgaqcaaaa@2DD1@ estimate alone could not be extrapolated to settings where prevalence and incidence of the STIs in question are different from the trial setting. However, efficacy estimates should be valid beyond the trial setting and enhance the interpretation and extrapolation of results. We have shown that efficacy estimation is feasible with Anderson-Gill analyses under realistic trial conditions. These analyses should be considered for HIV prevention trials in which the intervention may protect against both STI and HIV. This could be the case with female-use microbicides and diaphragms. The same methodological issues concerning statistical efficacy and effectiveness estimation and interpretation of results are posed for those trials.

Public health decision makers are unlikely to be interested in circumcision if effectiveness is low (< ~ 30%). On the basis of our findings, the usefulness of circumcision against HIV when effectiveness is high (> ~ 50%) may not be questioned since a small fraction of prevented HIV infections will be expected to be due to the indirect effect of circumcision against STI. It could be argued that separate estimates of efficacy against HIV and STI are most essential when effectiveness is moderate (~30%–50%), as public health decisions would have to give greater consideration to the role of STI in the prevention of HIV with MC. However, we argue that efficacy estimation of MC against STI and HIV is important in all cases because effectiveness estimates do not give the full picture for the longer-term community-level impact of MC. This is for two reasons. First, different combinations of efficacies (*E*_*H *_and *E*_*S*_) can produce equivalent effectiveness estimates in a trial context but not equivalent impact in a general population. Second, effectiveness estimated in a randomised trial does not correspond exactly to epidemiological effectiveness. In particular, the herd effect of MC benefiting females cannot be captured by trial estimates. By separately estimating *E*_*H *_and *E*_*S*_, transmission dynamic models can project better the potential epidemiological impact of MC. Further simulations with our model illustrated this point. For example, if MC had 40% efficacy against HIV and reached 75% of males in Kisumu, then after 10 years a 23% decline in HIV prevalence is possible in men compared to 7% in women, assuming MC does not protect against STI. However, if MC is also efficacious against STI, these declines in HIV prevalence are 29% for males and 14% in females, indicating the incremental benefit of STI efficacy is larger for women than men. This is essentially due to the fact that MC efficacy impact on male STI prevalence more rapidly than on HIV prevalence, and women's exposure to STI is consequently reduced quickly. Such insights would not be possible with effectiveness estimates alone. Thus, in terms of evaluating the public health potential of MC for both men and women, it will be essential to accurately estimate both *E*_*H *_and *E*_*S*_, even if high HIV effectiveness is observed.

Our mathematical model included only one simplistically modelled STI. The treatment of STI in this manner can be viewed as not one but a collection of STIs which taken together determine STI prevalence and collectively have an average HIV-STI interaction effect on HIV transmission. MC efficacy against these STIs can also be seen as some mean of the efficacies against the individual STIs. Though this is a simplified treatment of STIs, general conclusions on the level of importance of STIs held over a wide range of parameter assumptions in our sensitivity analyses.

Nevertheless, this still leaves unanswered the question of how to deal with several categories of STIs in a trial setting (e.g. genital ulcer disease, herpes, syphilis, chancroid, gonorrhoea, chlamydia, etc.). At one extreme, analyses could define STI-positive person-time on the basis of infection with any of the above and STI-negative person-time on basis of no STI infection of any type. At the other extreme, one could attempt to control individually for each of the different STIs in the statistical analyses, but how well this may work needs to be validated. If feasible, this would offer even greater value to the phase 3 trial by indicating the specific categories of STIs against which circumcision is beneficial.

## Abbreviations

*F *– effectiveness of male circumcision

F^
 MathType@MTEF@5@5@+=feaafiart1ev1aaatCvAUfKttLearuWrP9MDH5MBPbIqV92AaeXatLxBI9gBaebbnrfifHhDYfgasaacH8akY=wiFfYdH8Gipec8Eeeu0xXdbba9frFj0=OqFfea0dXdd9vqai=hGuQ8kuc9pgc9s8qqaq=dirpe0xb9q8qiLsFr0=vr0=vr0dc8meaabaqaciaacaGaaeqabaqabeGadaaakeaacuWGgbGrgaqcaaaa@2DD1@ – estimate of *F*

*E*_*H *_– efficacy of male circumcision against HIV

E^
 MathType@MTEF@5@5@+=feaafiart1ev1aaatCvAUfKttLearuWrP9MDH5MBPbIqV92AaeXatLxBI9gBaebbnrfifHhDYfgasaacH8akY=wiFfYdH8Gipec8Eeeu0xXdbba9frFj0=OqFfea0dXdd9vqai=hGuQ8kuc9pgc9s8qqaq=dirpe0xb9q8qiLsFr0=vr0=vr0dc8meaabaqaciaacaGaaeqabaqabeGadaaakeaacuWGfbqrgaqcaaaa@2DCF@_*H *_– estimate of *E*_*H*_

*E*_*S *_– efficacy of male circumcision against STI

E^
 MathType@MTEF@5@5@+=feaafiart1ev1aaatCvAUfKttLearuWrP9MDH5MBPbIqV92AaeXatLxBI9gBaebbnrfifHhDYfgasaacH8akY=wiFfYdH8Gipec8Eeeu0xXdbba9frFj0=OqFfea0dXdd9vqai=hGuQ8kuc9pgc9s8qqaq=dirpe0xb9q8qiLsFr0=vr0=vr0dc8meaabaqaciaacaGaaeqabaqabeGadaaakeaacuWGfbqrgaqcaaaa@2DCF@_*S *_– estimate of *E*_*S*_

*AF *– attributable fraction (fraction of HIV infections prevented due to MC efficacy against HIV)

HIV – human immunodeficiency virus

MC – male circumcision

STI – sexually transmitted infection

## Competing interests

The author(s) declare they have no competing interests

## Endnotes

### Stochastic mathematical model

**Figure 2:**
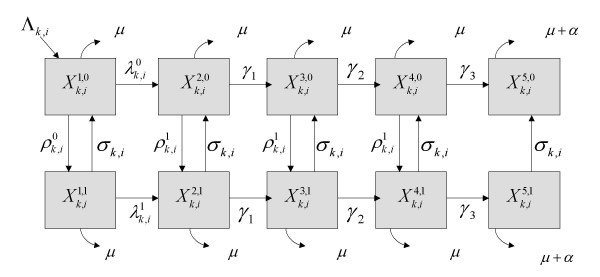
Stochastic compartmental mathematical model of phase III circumcision trial and HIV infection and co-circulating STI in the general population. Xk,ih,s
 MathType@MTEF@5@5@+=feaafiart1ev1aaatCvAUfKttLearuWrP9MDH5MBPbIqV92AaeXatLxBI9gBaebbnrfifHhDYfgasaacH8akY=wiFfYdH8Gipec8Eeeu0xXdbba9frFj0=OqFfea0dXdd9vqai=hGuQ8kuc9pgc9s8qqaq=dirpe0xb9q8qiLsFr0=vr0=vr0dc8meaabaqaciaacaGaaeqabaqabeGadaaakeaacqWGybawdaqhaaWcbaGaem4AaSMaeiilaWIaemyAaKgabaGaemiAaGMaeiilaWIaem4Camhaaaaa@3554@(*t*) represents the number of individuals in the general population at time t of sex k and sexual activity class i with HIV infection status h and STI status s. Flows indicate the 28 possible events.

The model consists of ten disease states (h = 1,...,5 and s = 1,2) representing five different stages of HIV infection and two states of STI infection (Figure 2). The number of individuals in the population in HIV infection state h, STI infection state s, activity class i and of sex k at time t, is given by Xk,ih,s
 MathType@MTEF@5@5@+=feaafiart1ev1aaatCvAUfKttLearuWrP9MDH5MBPbIqV92AaeXatLxBI9gBaebbnrfifHhDYfgasaacH8akY=wiFfYdH8Gipec8Eeeu0xXdbba9frFj0=OqFfea0dXdd9vqai=hGuQ8kuc9pgc9s8qqaq=dirpe0xb9q8qiLsFr0=vr0=vr0dc8meaabaqaciaacaGaaeqabaqabeGadaaakeaacqWGybawdaqhaaWcbaGaem4AaSMaeiilaWIaemyAaKgabaGaemiAaGMaeiilaWIaem4Camhaaaaa@3554@(*t*). HIV susceptibles are labelled with the superscript h = 1, full blown AIDS patients with h = 5, and HIV infecteds in three stages of HIV infection (having different degrees of infectiousness) with h = 2,3,4. Individuals infected with STI are denoted by s = 1 and those not infected with STI by s = 0. The sexually active population is stratified by sex (k = 1 for women, k = 2 for men) and by sexual activity class defined by the rate of sexual partner acquisition. Six activity classes are defined (i = 1,...,6) where at one extreme are individuals of low sexual activity (i = 1,2) and at the other are high activity individuals (i = 3,4,5,6). Transition events between states occur by infection, progression to disease, departure from the sexually active population or immigration into the population. Given a stratification of two sexes and six sexual activity classes, 336 possible events can occur (2 sexes × 6 classes × 28 possible events) in the absence a clinical trial, as defined in Figure 2 and Table 5.

**Table 5:** Events and flow rates of individuals between disease states in general population and clinical trials arms.

**Event r**	**Description of events for general population**	**Gross Rate (number individuals per year) R_r,k,i_(t)**
1	Recruitment into sexually active population	*R*_1,*k*,*i*_(*t*) = Λ_*k*,*i*_(*t*)
2	Departure from sexually active population (DFSA) of STI-/HIV- individuals	*R*_2,*k*,*i*_(*t*) = *μ *Xk,i1,0 MathType@MTEF@5@5@+=feaafiart1ev1aaatCvAUfKttLearuWrP9MDH5MBPbIqV92AaeXatLxBI9gBaebbnrfifHhDYfgasaacH8akY=wiFfYdH8Gipec8Eeeu0xXdbba9frFj0=OqFfea0dXdd9vqai=hGuQ8kuc9pgc9s8qqaq=dirpe0xb9q8qiLsFr0=vr0=vr0dc8meaabaqaciaacaGaaeqabaqabeGadaaakeaacqWGybawdaqhaaWcbaGaem4AaSMaeiilaWIaemyAaKgabaGaeGymaeJaeiilaWIaeGimaadaaaaa@346A@(*t*)
3	DFSA of STI-/HIV+ infecteds in first disease state	*R*_3,*k*,*i*_(*t*) = *μ *Xk,i2,0 MathType@MTEF@5@5@+=feaafiart1ev1aaatCvAUfKttLearuWrP9MDH5MBPbIqV92AaeXatLxBI9gBaebbnrfifHhDYfgasaacH8akY=wiFfYdH8Gipec8Eeeu0xXdbba9frFj0=OqFfea0dXdd9vqai=hGuQ8kuc9pgc9s8qqaq=dirpe0xb9q8qiLsFr0=vr0=vr0dc8meaabaqaciaacaGaaeqabaqabeGadaaakeaacqWGybawdaqhaaWcbaGaem4AaSMaeiilaWIaemyAaKgabaGaeGOmaiJaeiilaWIaeGimaadaaaaa@346C@(*t*)
4	DFSA of STI-/HIV+HIV infecteds in second disease state	*R*_4,*k*,*i*_(*t*) = *μ *Xk,i3,0 MathType@MTEF@5@5@+=feaafiart1ev1aaatCvAUfKttLearuWrP9MDH5MBPbIqV92AaeXatLxBI9gBaebbnrfifHhDYfgasaacH8akY=wiFfYdH8Gipec8Eeeu0xXdbba9frFj0=OqFfea0dXdd9vqai=hGuQ8kuc9pgc9s8qqaq=dirpe0xb9q8qiLsFr0=vr0=vr0dc8meaabaqaciaacaGaaeqabaqabeGadaaakeaacqWGybawdaqhaaWcbaGaem4AaSMaeiilaWIaemyAaKgabaGaeG4mamJaeiilaWIaeGimaadaaaaa@346E@(*t*)
5	DFSA of STI-/HIV+HIV infecteds in third disease state	*R*_5,*k*,*i*_(*t*) = *μ *Xk,i4,0 MathType@MTEF@5@5@+=feaafiart1ev1aaatCvAUfKttLearuWrP9MDH5MBPbIqV92AaeXatLxBI9gBaebbnrfifHhDYfgasaacH8akY=wiFfYdH8Gipec8Eeeu0xXdbba9frFj0=OqFfea0dXdd9vqai=hGuQ8kuc9pgc9s8qqaq=dirpe0xb9q8qiLsFr0=vr0=vr0dc8meaabaqaciaacaGaaeqabaqabeGadaaakeaacqWGybawdaqhaaWcbaGaem4AaSMaeiilaWIaemyAaKgabaGaeGinaqJaeiilaWIaeGimaadaaaaa@3470@(*t*)
6	Departure of AIDS patients who are STI-	*R*_6,*k*,*i*_(*t*) = (*μ *+ *α*)Xk,i5,0 MathType@MTEF@5@5@+=feaafiart1ev1aaatCvAUfKttLearuWrP9MDH5MBPbIqV92AaeXatLxBI9gBaebbnrfifHhDYfgasaacH8akY=wiFfYdH8Gipec8Eeeu0xXdbba9frFj0=OqFfea0dXdd9vqai=hGuQ8kuc9pgc9s8qqaq=dirpe0xb9q8qiLsFr0=vr0=vr0dc8meaabaqaciaacaGaaeqabaqabeGadaaakeaacqWGybawdaqhaaWcbaGaem4AaSMaeiilaWIaemyAaKgabaGaeGynauJaeiilaWIaeGimaadaaaaa@3472@(*t*)
7	HIV infection of STI-negative susceptibles	*R*_7,*k*,*i*_(*t*) = λk,i0 MathType@MTEF@5@5@+=feaafiart1ev1aaatCvAUfKttLearuWrP9MDH5MBPbIqV92AaeXatLxBI9gBaebbnrfifHhDYfgasaacH8akY=wiFfYdH8Gipec8Eeeu0xXdbba9frFj0=OqFfea0dXdd9vqai=hGuQ8kuc9pgc9s8qqaq=dirpe0xb9q8qiLsFr0=vr0=vr0dc8meaabaqaciaacaGaaeqabaqabeGadaaakeaaiiGacqWF7oaBdaqhaaWcbaGaem4AaSMaeiilaWIaemyAaKgabaGaeGimaadaaaaa@331C@(*t*)Xk,i1,0 MathType@MTEF@5@5@+=feaafiart1ev1aaatCvAUfKttLearuWrP9MDH5MBPbIqV92AaeXatLxBI9gBaebbnrfifHhDYfgasaacH8akY=wiFfYdH8Gipec8Eeeu0xXdbba9frFj0=OqFfea0dXdd9vqai=hGuQ8kuc9pgc9s8qqaq=dirpe0xb9q8qiLsFr0=vr0=vr0dc8meaabaqaciaacaGaaeqabaqabeGadaaakeaacqWGybawdaqhaaWcbaGaem4AaSMaeiilaWIaemyAaKgabaGaeGymaeJaeiilaWIaeGimaadaaaaa@346A@(*t*)
8	Progression to second state of HIV infection for STI-	*R*_8,*k*,*i*_(*t*) = *γ*_1_Xk,i2,0 MathType@MTEF@5@5@+=feaafiart1ev1aaatCvAUfKttLearuWrP9MDH5MBPbIqV92AaeXatLxBI9gBaebbnrfifHhDYfgasaacH8akY=wiFfYdH8Gipec8Eeeu0xXdbba9frFj0=OqFfea0dXdd9vqai=hGuQ8kuc9pgc9s8qqaq=dirpe0xb9q8qiLsFr0=vr0=vr0dc8meaabaqaciaacaGaaeqabaqabeGadaaakeaacqWGybawdaqhaaWcbaGaem4AaSMaeiilaWIaemyAaKgabaGaeGOmaiJaeiilaWIaeGimaadaaaaa@346C@(*t*)
9	Progression to third state of HIV infection for STI-	*R*_9,*k*,*i*_(*t*) = *γ*_2_Xk,i3,0 MathType@MTEF@5@5@+=feaafiart1ev1aaatCvAUfKttLearuWrP9MDH5MBPbIqV92AaeXatLxBI9gBaebbnrfifHhDYfgasaacH8akY=wiFfYdH8Gipec8Eeeu0xXdbba9frFj0=OqFfea0dXdd9vqai=hGuQ8kuc9pgc9s8qqaq=dirpe0xb9q8qiLsFr0=vr0=vr0dc8meaabaqaciaacaGaaeqabaqabeGadaaakeaacqWGybawdaqhaaWcbaGaem4AaSMaeiilaWIaemyAaKgabaGaeG4mamJaeiilaWIaeGimaadaaaaa@346E@(*t*)
10	Progression to AIDS for STI-	*R*_10,*k*,*i*_(*t*) = *γ*_3_Xk,i1,0 MathType@MTEF@5@5@+=feaafiart1ev1aaatCvAUfKttLearuWrP9MDH5MBPbIqV92AaeXatLxBI9gBaebbnrfifHhDYfgasaacH8akY=wiFfYdH8Gipec8Eeeu0xXdbba9frFj0=OqFfea0dXdd9vqai=hGuQ8kuc9pgc9s8qqaq=dirpe0xb9q8qiLsFr0=vr0=vr0dc8meaabaqaciaacaGaaeqabaqabeGadaaakeaacqWGybawdaqhaaWcbaGaem4AaSMaeiilaWIaemyAaKgabaGaeGymaeJaeiilaWIaeGimaadaaaaa@346A@(*t*)
11	Infection with STI for HIV- individuals	*R*_11,*k*,*i*_(*t*) = ρk,i0 MathType@MTEF@5@5@+=feaafiart1ev1aaatCvAUfKttLearuWrP9MDH5MBPbIqV92AaeXatLxBI9gBaebbnrfifHhDYfgasaacH8akY=wiFfYdH8Gipec8Eeeu0xXdbba9frFj0=OqFfea0dXdd9vqai=hGuQ8kuc9pgc9s8qqaq=dirpe0xb9q8qiLsFr0=vr0=vr0dc8meaabaqaciaacaGaaeqabaqabeGadaaakeaaiiGacqWFbpGCdaqhaaWcbaGaem4AaSMaeiilaWIaemyAaKgabaGaeGimaadaaaaa@3328@(*t*)Xk,i1,0 MathType@MTEF@5@5@+=feaafiart1ev1aaatCvAUfKttLearuWrP9MDH5MBPbIqV92AaeXatLxBI9gBaebbnrfifHhDYfgasaacH8akY=wiFfYdH8Gipec8Eeeu0xXdbba9frFj0=OqFfea0dXdd9vqai=hGuQ8kuc9pgc9s8qqaq=dirpe0xb9q8qiLsFr0=vr0=vr0dc8meaabaqaciaacaGaaeqabaqabeGadaaakeaacqWGybawdaqhaaWcbaGaem4AaSMaeiilaWIaemyAaKgabaGaeGymaeJaeiilaWIaeGimaadaaaaa@346A@(*t*)
12–14	Infection with STI for HIV+ individuals	*R*_*r*,*k*,*i*_(*t*) = ρk,i1 MathType@MTEF@5@5@+=feaafiart1ev1aaatCvAUfKttLearuWrP9MDH5MBPbIqV92AaeXatLxBI9gBaebbnrfifHhDYfgasaacH8akY=wiFfYdH8Gipec8Eeeu0xXdbba9frFj0=OqFfea0dXdd9vqai=hGuQ8kuc9pgc9s8qqaq=dirpe0xb9q8qiLsFr0=vr0=vr0dc8meaabaqaciaacaGaaeqabaqabeGadaaakeaaiiGacqWFbpGCdaqhaaWcbaGaem4AaSMaeiilaWIaemyAaKgabaGaeGymaedaaaaa@332A@(*t*)Xk,i..,0 MathType@MTEF@5@5@+=feaafiart1ev1aaatCvAUfKttLearuWrP9MDH5MBPbIqV92AaeXatLxBI9gBaebbnrfifHhDYfgasaacH8akY=wiFfYdH8Gipec8Eeeu0xXdbba9frFj0=OqFfea0dXdd9vqai=hGuQ8kuc9pgc9s8qqaq=dirpe0xb9q8qiLsFr0=vr0=vr0dc8meaabaqaciaacaGaaeqabaqabeGadaaakeaacqWGybawdaqhaaWcbaGaem4AaSMaeiilaWIaemyAaKgabaGaeiOla4IaeiOla4IaeiilaWIaeGimaadaaaaa@3542@(*t*)
15–19	Recovery from STI for HIV+ and HIV-	*R*_*r*,*k*,*i*_(*t*) = *σ*_*k*,*i*_(*t*)Xk,i..,1 MathType@MTEF@5@5@+=feaafiart1ev1aaatCvAUfKttLearuWrP9MDH5MBPbIqV92AaeXatLxBI9gBaebbnrfifHhDYfgasaacH8akY=wiFfYdH8Gipec8Eeeu0xXdbba9frFj0=OqFfea0dXdd9vqai=hGuQ8kuc9pgc9s8qqaq=dirpe0xb9q8qiLsFr0=vr0=vr0dc8meaabaqaciaacaGaaeqabaqabeGadaaakeaacqWGybawdaqhaaWcbaGaem4AaSMaeiilaWIaemyAaKgabaGaeiOla4IaeiOla4IaeiilaWIaeGymaedaaaaa@3544@(*t*)
20–23	DFSA for STI+	*R*_*r*,*k*,*i*_(*t*) = *μ *Xk,i..,1 MathType@MTEF@5@5@+=feaafiart1ev1aaatCvAUfKttLearuWrP9MDH5MBPbIqV92AaeXatLxBI9gBaebbnrfifHhDYfgasaacH8akY=wiFfYdH8Gipec8Eeeu0xXdbba9frFj0=OqFfea0dXdd9vqai=hGuQ8kuc9pgc9s8qqaq=dirpe0xb9q8qiLsFr0=vr0=vr0dc8meaabaqaciaacaGaaeqabaqabeGadaaakeaacqWGybawdaqhaaWcbaGaem4AaSMaeiilaWIaemyAaKgabaGaeiOla4IaeiOla4IaeiilaWIaeGymaedaaaaa@3544@(*t*)
24	Death rate of AIDS patients who are STI+	*R*_24,*k*,*i*_(*t*) = (*μ *+ *α*)Xk,i5,1 MathType@MTEF@5@5@+=feaafiart1ev1aaatCvAUfKttLearuWrP9MDH5MBPbIqV92AaeXatLxBI9gBaebbnrfifHhDYfgasaacH8akY=wiFfYdH8Gipec8Eeeu0xXdbba9frFj0=OqFfea0dXdd9vqai=hGuQ8kuc9pgc9s8qqaq=dirpe0xb9q8qiLsFr0=vr0=vr0dc8meaabaqaciaacaGaaeqabaqabeGadaaakeaacqWGybawdaqhaaWcbaGaem4AaSMaeiilaWIaemyAaKgabaGaeGynauJaeiilaWIaeGymaedaaaaa@3474@(*t*)
25	HIV infection of STI+ positive susceptibles	*R*_25,*k*,*i*_(*t*) = λk,i1 MathType@MTEF@5@5@+=feaafiart1ev1aaatCvAUfKttLearuWrP9MDH5MBPbIqV92AaeXatLxBI9gBaebbnrfifHhDYfgasaacH8akY=wiFfYdH8Gipec8Eeeu0xXdbba9frFj0=OqFfea0dXdd9vqai=hGuQ8kuc9pgc9s8qqaq=dirpe0xb9q8qiLsFr0=vr0=vr0dc8meaabaqaciaacaGaaeqabaqabeGadaaakeaaiiGacqWF7oaBdaqhaaWcbaGaem4AaSMaeiilaWIaemyAaKgabaGaeGymaedaaaaa@331E@(*t*)Xk,i1,1 MathType@MTEF@5@5@+=feaafiart1ev1aaatCvAUfKttLearuWrP9MDH5MBPbIqV92AaeXatLxBI9gBaebbnrfifHhDYfgasaacH8akY=wiFfYdH8Gipec8Eeeu0xXdbba9frFj0=OqFfea0dXdd9vqai=hGuQ8kuc9pgc9s8qqaq=dirpe0xb9q8qiLsFr0=vr0=vr0dc8meaabaqaciaacaGaaeqabaqabeGadaaakeaacqWGybawdaqhaaWcbaGaem4AaSMaeiilaWIaemyAaKgabaGaeGymaeJaeiilaWIaeGymaedaaaaa@346C@(*t*)
26	Progression to second state of HIV infection for STI+	*R*_26,*k*,*i*_(*t*) = *γ*_1_Xk,i2,1 MathType@MTEF@5@5@+=feaafiart1ev1aaatCvAUfKttLearuWrP9MDH5MBPbIqV92AaeXatLxBI9gBaebbnrfifHhDYfgasaacH8akY=wiFfYdH8Gipec8Eeeu0xXdbba9frFj0=OqFfea0dXdd9vqai=hGuQ8kuc9pgc9s8qqaq=dirpe0xb9q8qiLsFr0=vr0=vr0dc8meaabaqaciaacaGaaeqabaqabeGadaaakeaacqWGybawdaqhaaWcbaGaem4AaSMaeiilaWIaemyAaKgabaGaeGOmaiJaeiilaWIaeGymaedaaaaa@346E@(*t*)
27	Progression to third state of HIV infection for STI+	*R*_27,*k*,*i*_(*t*) = *γ*_2_Xk,i3,1 MathType@MTEF@5@5@+=feaafiart1ev1aaatCvAUfKttLearuWrP9MDH5MBPbIqV92AaeXatLxBI9gBaebbnrfifHhDYfgasaacH8akY=wiFfYdH8Gipec8Eeeu0xXdbba9frFj0=OqFfea0dXdd9vqai=hGuQ8kuc9pgc9s8qqaq=dirpe0xb9q8qiLsFr0=vr0=vr0dc8meaabaqaciaacaGaaeqabaqabeGadaaakeaacqWGybawdaqhaaWcbaGaem4AaSMaeiilaWIaemyAaKgabaGaeG4mamJaeiilaWIaeGymaedaaaaa@3470@(*t*)
28	Progression to AIDS for STI+	*R*_28,*k*,*i*_(*t*) = *γ*_3_Xk,i4,1 MathType@MTEF@5@5@+=feaafiart1ev1aaatCvAUfKttLearuWrP9MDH5MBPbIqV92AaeXatLxBI9gBaebbnrfifHhDYfgasaacH8akY=wiFfYdH8Gipec8Eeeu0xXdbba9frFj0=OqFfea0dXdd9vqai=hGuQ8kuc9pgc9s8qqaq=dirpe0xb9q8qiLsFr0=vr0=vr0dc8meaabaqaciaacaGaaeqabaqabeGadaaakeaacqWGybawdaqhaaWcbaGaem4AaSMaeiilaWIaemyAaKgabaGaeGinaqJaeiilaWIaeGymaedaaaaa@3472@(*t*)
	Description of events in control arm of trial	
29–55	Defined analogously to general population	
	Description of event for circumcised individuals	
56–82	Defined analogously to general population	
61	HIV infection of STI-negative susceptibles	R_61,*k*,*i*_(*t*) = (1-*RS*_*HIV*_)λk,i0 MathType@MTEF@5@5@+=feaafiart1ev1aaatCvAUfKttLearuWrP9MDH5MBPbIqV92AaeXatLxBI9gBaebbnrfifHhDYfgasaacH8akY=wiFfYdH8Gipec8Eeeu0xXdbba9frFj0=OqFfea0dXdd9vqai=hGuQ8kuc9pgc9s8qqaq=dirpe0xb9q8qiLsFr0=vr0=vr0dc8meaabaqaciaacaGaaeqabaqabeGadaaakeaaiiGacqWF7oaBdaqhaaWcbaGaem4AaSMaeiilaWIaemyAaKgabaGaeGimaadaaaaa@331C@(*t*)Zk,i1,0 MathType@MTEF@5@5@+=feaafiart1ev1aaatCvAUfKttLearuWrP9MDH5MBPbIqV92AaeXatLxBI9gBaebbnrfifHhDYfgasaacH8akY=wiFfYdH8Gipec8Eeeu0xXdbba9frFj0=OqFfea0dXdd9vqai=hGuQ8kuc9pgc9s8qqaq=dirpe0xb9q8qiLsFr0=vr0=vr0dc8meaabaqaciaacaGaaeqabaqabeGadaaakeaacqWGAbGwdaqhaaWcbaGaem4AaSMaeiilaWIaemyAaKgabaGaeGymaeJaeiilaWIaeGimaadaaaaa@346E@(*t*)
65	Infection with STI for HIV- individuals	R_65,*k*,*i*_(*t*) = (1-*RS*_*STI*_)ρk,i0 MathType@MTEF@5@5@+=feaafiart1ev1aaatCvAUfKttLearuWrP9MDH5MBPbIqV92AaeXatLxBI9gBaebbnrfifHhDYfgasaacH8akY=wiFfYdH8Gipec8Eeeu0xXdbba9frFj0=OqFfea0dXdd9vqai=hGuQ8kuc9pgc9s8qqaq=dirpe0xb9q8qiLsFr0=vr0=vr0dc8meaabaqaciaacaGaaeqabaqabeGadaaakeaaiiGacqWFbpGCdaqhaaWcbaGaem4AaSMaeiilaWIaemyAaKgabaGaeGimaadaaaaa@3328@(*t*)Zk,i1,0 MathType@MTEF@5@5@+=feaafiart1ev1aaatCvAUfKttLearuWrP9MDH5MBPbIqV92AaeXatLxBI9gBaebbnrfifHhDYfgasaacH8akY=wiFfYdH8Gipec8Eeeu0xXdbba9frFj0=OqFfea0dXdd9vqai=hGuQ8kuc9pgc9s8qqaq=dirpe0xb9q8qiLsFr0=vr0=vr0dc8meaabaqaciaacaGaaeqabaqabeGadaaakeaacqWGAbGwdaqhaaWcbaGaem4AaSMaeiilaWIaemyAaKgabaGaeGymaeJaeiilaWIaeGimaadaaaaa@346E@(*t*)
66	Infection with STI for HIV+ individuals	R_66,*k*,*i*_(*t*) = (1-*RS*_*STI*_)ρk,i0 MathType@MTEF@5@5@+=feaafiart1ev1aaatCvAUfKttLearuWrP9MDH5MBPbIqV92AaeXatLxBI9gBaebbnrfifHhDYfgasaacH8akY=wiFfYdH8Gipec8Eeeu0xXdbba9frFj0=OqFfea0dXdd9vqai=hGuQ8kuc9pgc9s8qqaq=dirpe0xb9q8qiLsFr0=vr0=vr0dc8meaabaqaciaacaGaaeqabaqabeGadaaakeaaiiGacqWFbpGCdaqhaaWcbaGaem4AaSMaeiilaWIaemyAaKgabaGaeGimaadaaaaa@3328@(*t*)Zk,i1,0 MathType@MTEF@5@5@+=feaafiart1ev1aaatCvAUfKttLearuWrP9MDH5MBPbIqV92AaeXatLxBI9gBaebbnrfifHhDYfgasaacH8akY=wiFfYdH8Gipec8Eeeu0xXdbba9frFj0=OqFfea0dXdd9vqai=hGuQ8kuc9pgc9s8qqaq=dirpe0xb9q8qiLsFr0=vr0=vr0dc8meaabaqaciaacaGaaeqabaqabeGadaaakeaacqWGAbGwdaqhaaWcbaGaem4AaSMaeiilaWIaemyAaKgabaGaeGymaeJaeiilaWIaeGimaadaaaaa@346E@(*t*)
79	HIV infection of STI+ positive susceptibles	R_79,*k*,*i*_(*t*) = (1-*RS*_*HIV*_)λk,i1 MathType@MTEF@5@5@+=feaafiart1ev1aaatCvAUfKttLearuWrP9MDH5MBPbIqV92AaeXatLxBI9gBaebbnrfifHhDYfgasaacH8akY=wiFfYdH8Gipec8Eeeu0xXdbba9frFj0=OqFfea0dXdd9vqai=hGuQ8kuc9pgc9s8qqaq=dirpe0xb9q8qiLsFr0=vr0=vr0dc8meaabaqaciaacaGaaeqabaqabeGadaaakeaaiiGacqWF7oaBdaqhaaWcbaGaem4AaSMaeiilaWIaemyAaKgabaGaeGymaedaaaaa@331E@(*t*)Zk,i1,1 MathType@MTEF@5@5@+=feaafiart1ev1aaatCvAUfKttLearuWrP9MDH5MBPbIqV92AaeXatLxBI9gBaebbnrfifHhDYfgasaacH8akY=wiFfYdH8Gipec8Eeeu0xXdbba9frFj0=OqFfea0dXdd9vqai=hGuQ8kuc9pgc9s8qqaq=dirpe0xb9q8qiLsFr0=vr0=vr0dc8meaabaqaciaacaGaaeqabaqabeGadaaakeaacqWGAbGwdaqhaaWcbaGaem4AaSMaeiilaWIaemyAaKgabaGaeGymaeJaeiilaWIaeGymaedaaaaa@3470@(*t*)

**Figure 3:**
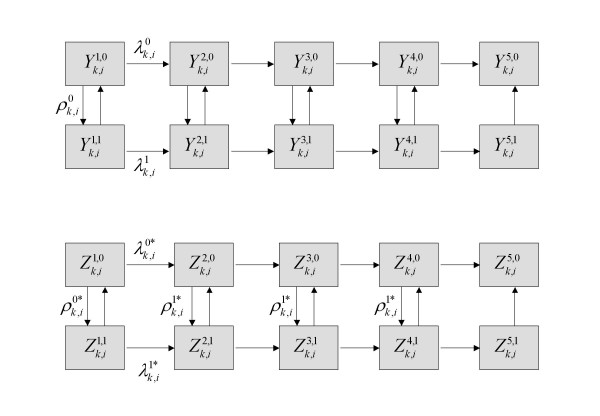
Additional compartments for clinical trial in stochastic compartmental mathematical model. Yk,ih,s
 MathType@MTEF@5@5@+=feaafiart1ev1aaatCvAUfKttLearuWrP9MDH5MBPbIqV92AaeXatLxBI9gBaebbnrfifHhDYfgasaacH8akY=wiFfYdH8Gipec8Eeeu0xXdbba9frFj0=OqFfea0dXdd9vqai=hGuQ8kuc9pgc9s8qqaq=dirpe0xb9q8qiLsFr0=vr0=vr0dc8meaabaqaciaacaGaaeqabaqabeGadaaakeaacqWGzbqwdaqhaaWcbaGaem4AaSMaeiilaWIaemyAaKgabaGaemiAaGMaeiilaWIaem4Camhaaaaa@3556@(*t*) is the number of individuals in the control arm of the trial and Zk,ih,s
 MathType@MTEF@5@5@+=feaafiart1ev1aaatCvAUfKttLearuWrP9MDH5MBPbIqV92AaeXatLxBI9gBaebbnrfifHhDYfgasaacH8akY=wiFfYdH8Gipec8Eeeu0xXdbba9frFj0=OqFfea0dXdd9vqai=hGuQ8kuc9pgc9s8qqaq=dirpe0xb9q8qiLsFr0=vr0=vr0dc8meaabaqaciaacaGaaeqabaqabeGadaaakeaacqWGAbGwdaqhaaWcbaGaem4AaSMaeiilaWIaemyAaKgabaGaemiAaGMaeiilaWIaem4Camhaaaaa@3558@(*t*) is the number in the treated (circumcised) arm. Circumcision acts to reduce the rate of HIV infection according to λk,i0*
 MathType@MTEF@5@5@+=feaafiart1ev1aaatCvAUfKttLearuWrP9MDH5MBPbIqV92AaeXatLxBI9gBaebbnrfifHhDYfgasaacH8akY=wiFfYdH8Gipec8Eeeu0xXdbba9frFj0=OqFfea0dXdd9vqai=hGuQ8kuc9pgc9s8qqaq=dirpe0xb9q8qiLsFr0=vr0=vr0dc8meaabaqaciaacaGaaeqabaqabeGadaaakeaaiiGacqWF7oaBdaqhaaWcbaGaem4AaSMaeiilaWIaemyAaKgabaGaeGimaaJaeiOkaOcaaaaa@33F8@(*t*) = (1 - *E*_*H*_)λk,i0
 MathType@MTEF@5@5@+=feaafiart1ev1aaatCvAUfKttLearuWrP9MDH5MBPbIqV92AaeXatLxBI9gBaebbnrfifHhDYfgasaacH8akY=wiFfYdH8Gipec8Eeeu0xXdbba9frFj0=OqFfea0dXdd9vqai=hGuQ8kuc9pgc9s8qqaq=dirpe0xb9q8qiLsFr0=vr0=vr0dc8meaabaqaciaacaGaaeqabaqabeGadaaakeaaiiGacqWF7oaBdaqhaaWcbaGaem4AaSMaeiilaWIaemyAaKgabaGaeGimaadaaaaa@331C@(*t*) and λk,i1*
 MathType@MTEF@5@5@+=feaafiart1ev1aaatCvAUfKttLearuWrP9MDH5MBPbIqV92AaeXatLxBI9gBaebbnrfifHhDYfgasaacH8akY=wiFfYdH8Gipec8Eeeu0xXdbba9frFj0=OqFfea0dXdd9vqai=hGuQ8kuc9pgc9s8qqaq=dirpe0xb9q8qiLsFr0=vr0=vr0dc8meaabaqaciaacaGaaeqabaqabeGadaaakeaaiiGacqWF7oaBdaqhaaWcbaGaem4AaSMaeiilaWIaemyAaKgabaGaeGymaeJaeiOkaOcaaaaa@33FA@(*t*) = (1 - *E*_*H*_)λk,i1
 MathType@MTEF@5@5@+=feaafiart1ev1aaatCvAUfKttLearuWrP9MDH5MBPbIqV92AaeXatLxBI9gBaebbnrfifHhDYfgasaacH8akY=wiFfYdH8Gipec8Eeeu0xXdbba9frFj0=OqFfea0dXdd9vqai=hGuQ8kuc9pgc9s8qqaq=dirpe0xb9q8qiLsFr0=vr0=vr0dc8meaabaqaciaacaGaaeqabaqabeGadaaakeaaiiGacqWF7oaBdaqhaaWcbaGaem4AaSMaeiilaWIaemyAaKgabaGaeGymaedaaaaa@331E@(*t*) and the rate of STI infection according to ρk,i0*
 MathType@MTEF@5@5@+=feaafiart1ev1aaatCvAUfKttLearuWrP9MDH5MBPbIqV92AaeXatLxBI9gBaebbnrfifHhDYfgasaacH8akY=wiFfYdH8Gipec8Eeeu0xXdbba9frFj0=OqFfea0dXdd9vqai=hGuQ8kuc9pgc9s8qqaq=dirpe0xb9q8qiLsFr0=vr0=vr0dc8meaabaqaciaacaGaaeqabaqabeGadaaakeaaiiGacqWFbpGCdaqhaaWcbaGaem4AaSMaeiilaWIaemyAaKgabaGaeGimaaJaeiOkaOcaaaaa@3404@(*t*) = (1 - *E*_*S*_)ρk,i0
 MathType@MTEF@5@5@+=feaafiart1ev1aaatCvAUfKttLearuWrP9MDH5MBPbIqV92AaeXatLxBI9gBaebbnrfifHhDYfgasaacH8akY=wiFfYdH8Gipec8Eeeu0xXdbba9frFj0=OqFfea0dXdd9vqai=hGuQ8kuc9pgc9s8qqaq=dirpe0xb9q8qiLsFr0=vr0=vr0dc8meaabaqaciaacaGaaeqabaqabeGadaaakeaaiiGacqWFbpGCdaqhaaWcbaGaem4AaSMaeiilaWIaemyAaKgabaGaeGimaadaaaaa@3328@(*t*) and ρk,i1*
 MathType@MTEF@5@5@+=feaafiart1ev1aaatCvAUfKttLearuWrP9MDH5MBPbIqV92AaeXatLxBI9gBaebbnrfifHhDYfgasaacH8akY=wiFfYdH8Gipec8Eeeu0xXdbba9frFj0=OqFfea0dXdd9vqai=hGuQ8kuc9pgc9s8qqaq=dirpe0xb9q8qiLsFr0=vr0=vr0dc8meaabaqaciaacaGaaeqabaqabeGadaaakeaaiiGacqWFbpGCdaqhaaWcbaGaem4AaSMaeiilaWIaemyAaKgabaGaeGymaeJaeiOkaOcaaaaa@3406@(*t*) = (1 - *E*_*S*_)ρk,i1
 MathType@MTEF@5@5@+=feaafiart1ev1aaatCvAUfKttLearuWrP9MDH5MBPbIqV92AaeXatLxBI9gBaebbnrfifHhDYfgasaacH8akY=wiFfYdH8Gipec8Eeeu0xXdbba9frFj0=OqFfea0dXdd9vqai=hGuQ8kuc9pgc9s8qqaq=dirpe0xb9q8qiLsFr0=vr0=vr0dc8meaabaqaciaacaGaaeqabaqabeGadaaakeaaiiGacqWFbpGCdaqhaaWcbaGaem4AaSMaeiilaWIaemyAaKgabaGaeGymaedaaaaa@332A@(*t*) where *E*_*H *_and *E*_*S *_are the efficacy of circumcision against HIV and STI.

Upon commencement of the clinical trial in the defined population, a sample of individuals is randomized between control and treated arms. As defined in Figure 3, the number of controls of HIV disease state h, STI infection state s, sex k and activity class i at time t is denoted Yk,ih,s
 MathType@MTEF@5@5@+=feaafiart1ev1aaatCvAUfKttLearuWrP9MDH5MBPbIqV92AaeXatLxBI9gBaebbnrfifHhDYfgasaacH8akY=wiFfYdH8Gipec8Eeeu0xXdbba9frFj0=OqFfea0dXdd9vqai=hGuQ8kuc9pgc9s8qqaq=dirpe0xb9q8qiLsFr0=vr0=vr0dc8meaabaqaciaacaGaaeqabaqabeGadaaakeaacqWGzbqwdaqhaaWcbaGaem4AaSMaeiilaWIaemyAaKgabaGaemiAaGMaeiilaWIaem4Camhaaaaa@3556@(*t*). The number circumcised at time t of disease state h, state i, sex k and activity class i is given by Zk,ih,s
 MathType@MTEF@5@5@+=feaafiart1ev1aaatCvAUfKttLearuWrP9MDH5MBPbIqV92AaeXatLxBI9gBaebbnrfifHhDYfgasaacH8akY=wiFfYdH8Gipec8Eeeu0xXdbba9frFj0=OqFfea0dXdd9vqai=hGuQ8kuc9pgc9s8qqaq=dirpe0xb9q8qiLsFr0=vr0=vr0dc8meaabaqaciaacaGaaeqabaqabeGadaaakeaacqWGAbGwdaqhaaWcbaGaem4AaSMaeiilaWIaemyAaKgabaGaemiAaGMaeiilaWIaem4Camhaaaaa@3558@(*t*). The number of possible events after the trial begins is 984 (2 sexes × 6 activity classes × 82 events), as given in Table 5.

In the stochastic simulations, a random sequence of individual events is generated where each of the 984 possible events occurs with probability Pr,k,i(t)=Rr,k,i(t)/S(t)
 MathType@MTEF@5@5@+=feaafiart1ev1aaatCvAUfKttLearuWrP9MDH5MBPbIqV92AaeXatLxBI9gBaebbnrfifHhDYfgasaacH8akY=wiFfYdH8Gipec8Eeeu0xXdbba9frFj0=OqFfea0dXdd9vqai=hGuQ8kuc9pgc9s8qqaq=dirpe0xb9q8qiLsFr0=vr0=vr0dc8meaabaqaciaacaGaaeqabaqabeGadaaakeaadaWfqaqaaiabdcfaqbWcbaGaemOCaiNaeiilaWIaem4AaSMaeiilaWIaemyAaKgabeaakiabcIcaOiabdsha0jabcMcaPiabg2da9maaxababaGaemOuaifaleaacqWGYbGCcqGGSaalcqWGRbWAcqGGSaalcqWGPbqAaeqaaOGaeiikaGIaemiDaqNaeiykaKIaei4la8Iaem4uamLaeiikaGIaemiDaqNaeiykaKcaaa@47DA@ where S(t)=∑r,k,iRr,k,i(t)
 MathType@MTEF@5@5@+=feaafiart1ev1aaatCvAUfKttLearuWrP9MDH5MBPbIqV92AaeXatLxBI9gBaebbnrfifHhDYfgasaacH8akY=wiFfYdH8Gipec8Eeeu0xXdbba9frFj0=OqFfea0dXdd9vqai=hGuQ8kuc9pgc9s8qqaq=dirpe0xb9q8qiLsFr0=vr0=vr0dc8meaabaqaciaacaGaaeqabaqabeGadaaakeaacqWGtbWucqGGOaakcqWG0baDcqGGPaqkcqGH9aqpdaaeqbqaamaaxababaGaemOuaifaleaacqWGYbGCcqGGSaalcqWGRbWAcqGGSaalcqWGPbqAaeqaaOGaeiikaGIaemiDaqNaeiykaKcaleaacqWGYbGCcqGGSaalcqWGRbWAcqGGSaalcqWGPbqAaeqaniabggHiLdaaaa@4488@. The event chosen at each step of the sequence is determined by a random number generator according to the 984 probabilities. The time a person of sex k and class i spends in a specific state r before making a transition is assumed to be exponentially distributed with mean 1/Rr,k,i(t)
 MathType@MTEF@5@5@+=feaafiart1ev1aaatCvAUfKttLearuWrP9MDH5MBPbIqV92AaeXatLxBI9gBaebbnrfifHhDYfgasaacH8akY=wiFfYdH8Gipec8Eeeu0xXdbba9frFj0=OqFfea0dXdd9vqai=hGuQ8kuc9pgc9s8qqaq=dirpe0xb9q8qiLsFr0=vr0=vr0dc8meaabaqaciaacaGaaeqabaqabeGadaaakeaacqaIXaqmcqGGVaWldaWfqaqaaiabdkfasbWcbaGaemOCaiNaeiilaWIaem4AaSMaeiilaWIaemyAaKgabeaakiabcIcaOiabdsha0jabcMcaPaaa@38FC@. Furthermore, the time between any two events is exponentially distributed with mean *S*^-1^(*t*). The time of occurrence s of chosen event r can be determined by choosing a random number from a uniform distribution and setting it equal to F(s) in the equation *F*(*s*) = 1-exp(-*S*(*t*)*s*) and solving for s. Thus by an iterative process, a sequence of events and their time of occurrence is generated. With each new event all rates including forces of infection are automatically updated.

Key event flows in the model include force of HIV infection (λk,i0
 MathType@MTEF@5@5@+=feaafiart1ev1aaatCvAUfKttLearuWrP9MDH5MBPbIqV92AaeXatLxBI9gBaebbnrfifHhDYfgasaacH8akY=wiFfYdH8Gipec8Eeeu0xXdbba9frFj0=OqFfea0dXdd9vqai=hGuQ8kuc9pgc9s8qqaq=dirpe0xb9q8qiLsFr0=vr0=vr0dc8meaabaqaciaacaGaaeqabaqabeGadaaakeaaiiGacqWF7oaBdaqhaaWcbaGaem4AaSMaeiilaWIaemyAaKgabaGaeGimaadaaaaa@331C@(*t*) and λk,i1
 MathType@MTEF@5@5@+=feaafiart1ev1aaatCvAUfKttLearuWrP9MDH5MBPbIqV92AaeXatLxBI9gBaebbnrfifHhDYfgasaacH8akY=wiFfYdH8Gipec8Eeeu0xXdbba9frFj0=OqFfea0dXdd9vqai=hGuQ8kuc9pgc9s8qqaq=dirpe0xb9q8qiLsFr0=vr0=vr0dc8meaabaqaciaacaGaaeqabaqabeGadaaakeaaiiGacqWF7oaBdaqhaaWcbaGaem4AaSMaeiilaWIaemyAaKgabaGaeGymaedaaaaa@331E@(*t*)) and STI infection (ρk,i0
 MathType@MTEF@5@5@+=feaafiart1ev1aaatCvAUfKttLearuWrP9MDH5MBPbIqV92AaeXatLxBI9gBaebbnrfifHhDYfgasaacH8akY=wiFfYdH8Gipec8Eeeu0xXdbba9frFj0=OqFfea0dXdd9vqai=hGuQ8kuc9pgc9s8qqaq=dirpe0xb9q8qiLsFr0=vr0=vr0dc8meaabaqaciaacaGaaeqabaqabeGadaaakeaaiiGacqWFbpGCdaqhaaWcbaGaem4AaSMaeiilaWIaemyAaKgabaGaeGimaadaaaaa@3328@(*t*) and ρk,i1
 MathType@MTEF@5@5@+=feaafiart1ev1aaatCvAUfKttLearuWrP9MDH5MBPbIqV92AaeXatLxBI9gBaebbnrfifHhDYfgasaacH8akY=wiFfYdH8Gipec8Eeeu0xXdbba9frFj0=OqFfea0dXdd9vqai=hGuQ8kuc9pgc9s8qqaq=dirpe0xb9q8qiLsFr0=vr0=vr0dc8meaabaqaciaacaGaaeqabaqabeGadaaakeaaiiGacqWFbpGCdaqhaaWcbaGaem4AaSMaeiilaWIaemyAaKgabaGaeGymaedaaaaa@332A@(*t*)). The effect of treatment is assumed to reduce these forces of infection by an amount EffHIV and EffSTI, respectively. The force of HIV infection for sex k and activity class i at time t in the general population and controls is given by:

λk,i0(t)=mk,i(t)∑j=16{ϕk,i,j(t)[∑h=24βk*,j,ih−1(Xk*,jh,0(t)+Yk*,jh,0(t)+Zk*,jh,0(t))NAk*,j(t)]+ϕk,i,j(t)[∑h=24βk*,j,ih−1(Xk*,jh,1(t)+Yk*,jh,1(t)+Zk*,jh,1(t))NAk*,j(t)]a1}
 MathType@MTEF@5@5@+=feaafiart1ev1aaatCvAUfKttLearuWrP9MDH5MBPbIqV92AaeXatLxBI9gBaebbnrfifHhDYfgasaacH8akY=wiFfYdH8Gipec8Eeeu0xXdbba9frFj0=OqFfea0dXdd9vqai=hGuQ8kuc9pgc9s8qqaq=dirpe0xb9q8qiLsFr0=vr0=vr0dc8meaabaqaciaacaGaaeqabaqabeGadaaakeaafaqabeGabaaabaacciGae83UdW2aa0baaSqaaiabdUgaRjabcYcaSiabdMgaPbqaaiabicdaWaaakiabcIcaOiabdsha0jabcMcaPiabg2da9iabd2gaTnaaBaaaleaacqWGRbWAcqGGSaalcqWGPbqAaeqaaOGaeiikaGIaemiDaqNaeiykaKYaaabCaeaadaGabeqaaiab=v9aQnaaBaaaleaacqWGRbWAcqGGSaalcqWGPbqAcqGGSaalcqWGQbGAaeqaaOGaeiikaGIaemiDaqNaeiykaKYaamWaaeaadaWcaaqaamaaqadabaGae8NSdi2aa0baaSqaaiabdUgaRjabcQcaQiabcYcaSiabdQgaQjabcYcaSiabdMgaPbqaaiabdIgaOjabgkHiTiabigdaXaaakmaabmaabaGaemiwaG1aa0baaSqaaiabdUgaRjabcQcaQiabcYcaSiabdQgaQbqaaiabdIgaOjabcYcaSiabicdaWaaakiabcIcaOiabdsha0jabcMcaPiabgUcaRiabdMfaznaaDaaaleaacqWGRbWAcqGGQaGkcqGGSaalcqWGQbGAaeaacqWGObaAcqGGSaalcqaIWaamaaGccqGGOaakcqWG0baDcqGGPaqkcqGHRaWkcqWGAbGwdaqhaaWcbaGaem4AaSMaeiOkaOIaeiilaWIaemOAaOgabaGaemiAaGMaeiilaWIaeGimaadaaOGaeiikaGIaemiDaqNaeiykaKcacaGLOaGaayzkaaaaleaacqWGObaAcqGH9aqpcqaIYaGmaeaacqaI0aana0GaeyyeIuoaaOqaaiabd6eaojabdgeabnaaBaaaleaacqWGRbWAcqGGQaGkcqGGSaalcqWGQbGAaeqaaOGaeiikaGIaemiDaqNaeiykaKcaaaGaay5waiaaw2faaaGaay5EaaGaey4kaScaleaacqWGQbGAcqGH9aqpcqaIXaqmaeaacqaI2aGna0GaeyyeIuoaaOqaamaaciqabaGae8x1dO2aaSbaaSqaaiabdUgaRjabcYcaSiabdMgaPjabcYcaSiabdQgaQbqabaGccqGGOaakcqWG0baDcqGGPaqkdaWadaqaamaalaaabaWaaabmaeaacqWFYoGydaqhaaWcbaGaem4AaSMaeiOkaOIaeiilaWIaemOAaOMaeiilaWIaemyAaKgabaGaemiAaGMaeyOeI0IaeGymaedaaOWaaeWaaeaacqWGybawdaqhaaWcbaGaem4AaSMaeiOkaOIaeiilaWIaemOAaOgabaGaemiAaGMaeiilaWIaeGymaedaaOGaeiikaGIaemiDaqNaeiykaKIaey4kaSIaemywaK1aa0baaSqaaiabdUgaRjabcQcaQiabcYcaSiabdQgaQbqaaiabdIgaOjabcYcaSiabigdaXaaakiabcIcaOiabdsha0jabcMcaPiabgUcaRiabdQfaAnaaDaaaleaacqWGRbWAcqGGQaGkcqGGSaalcqWGQbGAaeaacqWGObaAcqGGSaalcqaIXaqmaaGccqGGOaakcqWG0baDcqGGPaqkaiaawIcacaGLPaaaaSqaaiabdIgaOjabg2da9iabikdaYaqaaiabisda0aqdcqGHris5aaGcbaGaemOta4Kaemyqae0aaSbaaSqaaiabdUgaRjabcQcaQiabcYcaSiabdQgaQbqabaGccqGGOaakcqWG0baDcqGGPaqkaaaacaGLBbGaayzxaaGaemyyae2aaSbaaSqaaiabigdaXaqabaaakiaaw2haaaaaaaa@EDC1@

Here m_k,i _(t) is the annual rate of partner acquisition of persons of sex k and class i, and βk∗,j,ip
 MathType@MTEF@5@5@+=feaafiart1ev1aaatCvAUfKttLearuWrP9MDH5MBPbIqV92AaeXatLxBI9gBaebbnrfifHhDYfgasaacH8akY=wiFfYdH8Gipec8Eeeu0xXdbba9frFj0=OqFfea0dXdd9vqai=hGuQ8kuc9pgc9s8qqaq=dirpe0xb9q8qiLsFr0=vr0=vr0dc8meaabaqaciaacaGaaeqabaqabeGadaaakeaaiiGacqWFYoGydaqhaaWcbaGaem4AaS2aaWbaaWqabeaacqGHxiIkaaWccqGGSaalcqWGQbGAcqGGSaalcqWGPbqAaeaacqWGWbaCaaaaaa@36E9@ is the per partnership HIV transmission probability from a person in HIV infection phase p and sex k* and class j to opposite sex k and class i. The term *φ*_*k*,*i*,*j*_(*t*) describes the mixing matrix elements. This is essentially the probability that an individual of sex k and class i chooses a partner of opposite sex k* and class j (see Desai et al [27] for more details on mixing equations). NA_k*,j_(t) is the total sexually active population of sex k* and class j. The term a1 is a multiplicative factor increasing the probability of transmission of HIV when the HIV infected partner is also infected with STI. Thus, λk,i0
 MathType@MTEF@5@5@+=feaafiart1ev1aaatCvAUfKttLearuWrP9MDH5MBPbIqV92AaeXatLxBI9gBaebbnrfifHhDYfgasaacH8akY=wiFfYdH8Gipec8Eeeu0xXdbba9frFj0=OqFfea0dXdd9vqai=hGuQ8kuc9pgc9s8qqaq=dirpe0xb9q8qiLsFr0=vr0=vr0dc8meaabaqaciaacaGaaeqabaqabeGadaaakeaaiiGacqWF7oaBdaqhaaWcbaGaem4AaSMaeiilaWIaemyAaKgabaGaeGimaadaaaaa@331C@(*t*) is a function of the rate of sexual partner change, the HIV transmission probability, HIV prevalence and epidemiological interaction between HIV and STI.

The force of HIV infection in an STI infected individual is λk,i1
 MathType@MTEF@5@5@+=feaafiart1ev1aaatCvAUfKttLearuWrP9MDH5MBPbIqV92AaeXatLxBI9gBaebbnrfifHhDYfgasaacH8akY=wiFfYdH8Gipec8Eeeu0xXdbba9frFj0=OqFfea0dXdd9vqai=hGuQ8kuc9pgc9s8qqaq=dirpe0xb9q8qiLsFr0=vr0=vr0dc8meaabaqaciaacaGaaeqabaqabeGadaaakeaaiiGacqWF7oaBdaqhaaWcbaGaem4AaSMaeiilaWIaemyAaKgabaGaeGymaedaaaaa@331E@(*t*) = *a*_2_λk,i0
 MathType@MTEF@5@5@+=feaafiart1ev1aaatCvAUfKttLearuWrP9MDH5MBPbIqV92AaeXatLxBI9gBaebbnrfifHhDYfgasaacH8akY=wiFfYdH8Gipec8Eeeu0xXdbba9frFj0=OqFfea0dXdd9vqai=hGuQ8kuc9pgc9s8qqaq=dirpe0xb9q8qiLsFr0=vr0=vr0dc8meaabaqaciaacaGaaeqabaqabeGadaaakeaaiiGacqWF7oaBdaqhaaWcbaGaem4AaSMaeiilaWIaemyAaKgabaGaeGimaadaaaaa@331C@(*t*), where a_2 _is a multiplicative factor increasing the probability of transmission of HIV when the HIV susceptible is infected with STI. Values for the above parameters used in the simulations are given in Table 1.

The force of HIV infection for the circumcised group is given by λk,i0*
 MathType@MTEF@5@5@+=feaafiart1ev1aaatCvAUfKttLearuWrP9MDH5MBPbIqV92AaeXatLxBI9gBaebbnrfifHhDYfgasaacH8akY=wiFfYdH8Gipec8Eeeu0xXdbba9frFj0=OqFfea0dXdd9vqai=hGuQ8kuc9pgc9s8qqaq=dirpe0xb9q8qiLsFr0=vr0=vr0dc8meaabaqaciaacaGaaeqabaqabeGadaaakeaaiiGacqWF7oaBdaqhaaWcbaGaem4AaSMaeiilaWIaemyAaKgabaGaeGimaaJaeiOkaOcaaaaa@33F8@(*t*) = (1 - *E*_*H*_)λk,i0
 MathType@MTEF@5@5@+=feaafiart1ev1aaatCvAUfKttLearuWrP9MDH5MBPbIqV92AaeXatLxBI9gBaebbnrfifHhDYfgasaacH8akY=wiFfYdH8Gipec8Eeeu0xXdbba9frFj0=OqFfea0dXdd9vqai=hGuQ8kuc9pgc9s8qqaq=dirpe0xb9q8qiLsFr0=vr0=vr0dc8meaabaqaciaacaGaaeqabaqabeGadaaakeaaiiGacqWF7oaBdaqhaaWcbaGaem4AaSMaeiilaWIaemyAaKgabaGaeGimaadaaaaa@331C@(*t*) and λk,i1*
 MathType@MTEF@5@5@+=feaafiart1ev1aaatCvAUfKttLearuWrP9MDH5MBPbIqV92AaeXatLxBI9gBaebbnrfifHhDYfgasaacH8akY=wiFfYdH8Gipec8Eeeu0xXdbba9frFj0=OqFfea0dXdd9vqai=hGuQ8kuc9pgc9s8qqaq=dirpe0xb9q8qiLsFr0=vr0=vr0dc8meaabaqaciaacaGaaeqabaqabeGadaaakeaaiiGacqWF7oaBdaqhaaWcbaGaem4AaSMaeiilaWIaemyAaKgabaGaeGymaeJaeiOkaOcaaaaa@33FA@(*t*) = (1 - *E*_*H*_)λk,i1
 MathType@MTEF@5@5@+=feaafiart1ev1aaatCvAUfKttLearuWrP9MDH5MBPbIqV92AaeXatLxBI9gBaebbnrfifHhDYfgasaacH8akY=wiFfYdH8Gipec8Eeeu0xXdbba9frFj0=OqFfea0dXdd9vqai=hGuQ8kuc9pgc9s8qqaq=dirpe0xb9q8qiLsFr0=vr0=vr0dc8meaabaqaciaacaGaaeqabaqabeGadaaakeaaiiGacqWF7oaBdaqhaaWcbaGaem4AaSMaeiilaWIaemyAaKgabaGaeGymaedaaaaa@331E@(*t*), where *E*_*H *_is the reduction in susceptibility to HIV due to circumcision.

The force of STI infection in HIV susceptibles of sex k and class i is given by:

ρk,i0(t)=mk,i(t)∑j=16ξk*,j,iϕk,i,j(t)[∑h=24(Xk*,jh,1(t)+Yk*,jh,1(t)+Zk*,jh,1(t))NAk*,j(t)b1+Xk*,j1,1(t)+Yk*,j1,1(t)+Zk*,j1,1(t)NAk*,j(t)].
 MathType@MTEF@5@5@+=feaafiart1ev1aaatCvAUfKttLearuWrP9MDH5MBPbIqV92AaeXatLxBI9gBaebbnrfifHhDYfgasaacH8akY=wiFfYdH8Gipec8Eeeu0xXdbba9frFj0=OqFfea0dXdd9vqai=hGuQ8kuc9pgc9s8qqaq=dirpe0xb9q8qiLsFr0=vr0=vr0dc8meaabaqaciaacaGaaeqabaqabeGadaaakeaafaqabeGabaaabaacciGamWiG=f8aYnacmcyhaaWcbGaJakadmc4GRbWAcWaJakilaWIamWiGdMgaPbqaiWiGcWaJaIimaadaaOGamWiGcIcaOiadmc4G0baDcWaJakykaKIamWiGg2da9iadmc4GTbqBdGaJaUbaaSqaiWiGcWaJao4AaSMamWiGcYcaSiadmc4GPbqAaeqcmciakiadmcOGOaakcWaJaoiDaqNamWiGcMcaPmacmcieWbqaiWiGcWaJa+NVdG3aiWiGBaaaleacmcOamWiGdUgaRjadmcOGQaGkcWaJakilaWIamWiGdQgaQjadmcOGSaalcWaJaoyAaKgabKaJacGccWaJa+x1dO2aiWiGBaaaleacmcOamWiGdUgaRjadmcOGSaalcWaJaoyAaKMamWiGcYcaSiadmc4GQbGAaeqcmciakiadmcOGOaakcWaJaoiDaqNamWiGcMcaPmacmc4abaqaiWiGdGaJaUaaaeacmc4aiWiGqadabGaJaoacmcyadaqaiWiGcWaJaoiwaG1aiWiGDaaaleacmcOamWiGdUgaRjadmcOGQaGkcWaJakilaWIamWiGdQgaQbqaiWiGcWaJaoiAaGMamWiGcYcaSiadmciIXaqmaaGccWaJakikaGIamWiGdsha0jadmcOGPaqkcWaJaA4kaSIamWiGdMfaznacmcyhaaWcbGaJakadmc4GRbWAcWaJakOkaOIamWiGcYcaSiadmc4GQbGAaeacmcOamWiGdIgaOjadmcOGSaalcWaJaIymaedaaOGamWiGcIcaOiadmc4G0baDcWaJakykaKIamWiGgUcaRiadmc4GAbGwdGaJa2baaSqaiWiGcWaJao4AaSMamWiGcQcaQiadmcOGSaalcWaJaoOAaOgabGaJakadmc4GObaAcWaJakilaWIamWiGigdaXaaakiadmcOGOaakcWaJaoiDaqNamWiGcMcaPaGaiWiGwIcacGaJaAzkaaaaleacmcOamWiGdIgaOjadmcOH9aqpcWaJaIOmaidabGaJakadmciI0aana0GamWiGggHiLdaakeacmcOamWiGd6eaojadmc4GbbqqdGaJaUbaaSqaiWiGcWaJao4AaSMamWiGcQcaQiadmcOGSaalcWaJaoOAaOgabKaJacGccWaJakikaGIamWiGdsha0jadmcOGPaqkaaaacGaJaA5waaGamWiGdkgaInacmc4gaaWcbGaJakadmciIXaqmaeqcmciakiadmcOHRaWkaSqaiWiGcWaJaoOAaOMamWiGg2da9iadmciIXaqmaeacmcOamWiGiAda2aqdcWaJaAyeIuoaaOqaamaadiaabaWaaSaaaeaacWaJaoiwaG1aiWiGDaaaleacmcOamWiGdUgaRjadmcOGQaGkcWaJakilaWIamWiGdQgaQbqaiWiGcWaJaIymaeJamWiGcYcaSiadmciIXaqmaaGccWaJakikaGIamWiGdsha0jadmcOGPaqkcWaJaA4kaSIamWiGdMfaznacmcyhaaWcbGaJakadmc4GRbWAcWaJakOkaOIamWiGcYcaSiadmc4GQbGAaeacmcOamWiGigdaXiadmcOGSaalcWaJaIymaedaaOGamWiGcIcaOiadmc4G0baDcWaJakykaKIamWiGgUcaRiadmc4GAbGwdGaJa2baaSqaiWiGcWaJao4AaSMamWiGcQcaQiadmcOGSaalcWaJaoOAaOgabGaJakadmciIXaqmcWaJakilaWIamWiGigdaXaaakiadmcOGOaakcWaJaoiDaqNamWiGcMcaPaqaaiadmc4GobGtcWaJaoyqae0aiWiGBaaaleacmcOamWiGdUgaRjadmcOGQaGkcWaJakilaWIamWiGdQgaQbqajWiGaOGamWiGcIcaOiadmc4G0baDcWaJakykaKcaaaGaayzxaaGaeiOla4caaaaa@948E@

Here *ξ*_k*,*j*,*i *_is the per partnership transmission probability of STI from sex k* and sexual activity class j to opposite sex k and class i. Unlike for HIV transmission probabilities, the values for STI transmission probabilities do not change by sexual activity class. We have chosen these values because STI transmission probabilities for bacterial STIs are high and are less affected by number of sex acts within a partnership. In addition, we are not trying to represent the exact biology of a given STI, and the STI needs to reflect a number of different aetiologies. The parameter b1 is a multiplicative factor increasing the probability of STI transmission when the STI-positive partner is also HIV positive. In our model we set b_1 _= b_2 _= 1.

The force of STI infection in a HIV positive individual of sex k and class i is given by ρk,i1
 MathType@MTEF@5@5@+=feaafiart1ev1aaatCvAUfKttLearuWrP9MDH5MBPbIqV92AaeXatLxBI9gBaebbnrfifHhDYfgasaacH8akY=wiFfYdH8Gipec8Eeeu0xXdbba9frFj0=OqFfea0dXdd9vqai=hGuQ8kuc9pgc9s8qqaq=dirpe0xb9q8qiLsFr0=vr0=vr0dc8meaabaqaciaacaGaaeqabaqabeGadaaakeaaiiGacqWFbpGCdaqhaaWcbaGaem4AaSMaeiilaWIaemyAaKgabaGaeGymaedaaaaa@332A@(*t*) = *b*_2_ρk,i0
 MathType@MTEF@5@5@+=feaafiart1ev1aaatCvAUfKttLearuWrP9MDH5MBPbIqV92AaeXatLxBI9gBaebbnrfifHhDYfgasaacH8akY=wiFfYdH8Gipec8Eeeu0xXdbba9frFj0=OqFfea0dXdd9vqai=hGuQ8kuc9pgc9s8qqaq=dirpe0xb9q8qiLsFr0=vr0=vr0dc8meaabaqaciaacaGaaeqabaqabeGadaaakeaaiiGacqWFbpGCdaqhaaWcbaGaem4AaSMaeiilaWIaemyAaKgabaGaeGimaadaaaaa@3328@(*t*), where b_2 _is a multiplicative factor increasing the probability of STI transmission when the STI susceptible individual is HIV positive. The force of STI infection for the circumcised group is given by ρk,i0*
 MathType@MTEF@5@5@+=feaafiart1ev1aaatCvAUfKttLearuWrP9MDH5MBPbIqV92AaeXatLxBI9gBaebbnrfifHhDYfgasaacH8akY=wiFfYdH8Gipec8Eeeu0xXdbba9frFj0=OqFfea0dXdd9vqai=hGuQ8kuc9pgc9s8qqaq=dirpe0xb9q8qiLsFr0=vr0=vr0dc8meaabaqaciaacaGaaeqabaqabeGadaaakeaaiiGacqWFbpGCdaqhaaWcbaGaem4AaSMaeiilaWIaemyAaKgabaGaeGimaaJaeiOkaOcaaaaa@3404@(*t*) = (1 - *E*_*S*_)ρk,i0
 MathType@MTEF@5@5@+=feaafiart1ev1aaatCvAUfKttLearuWrP9MDH5MBPbIqV92AaeXatLxBI9gBaebbnrfifHhDYfgasaacH8akY=wiFfYdH8Gipec8Eeeu0xXdbba9frFj0=OqFfea0dXdd9vqai=hGuQ8kuc9pgc9s8qqaq=dirpe0xb9q8qiLsFr0=vr0=vr0dc8meaabaqaciaacaGaaeqabaqabeGadaaakeaaiiGacqWFbpGCdaqhaaWcbaGaem4AaSMaeiilaWIaemyAaKgabaGaeGimaadaaaaa@3328@(*t*) and ρk,i1*
 MathType@MTEF@5@5@+=feaafiart1ev1aaatCvAUfKttLearuWrP9MDH5MBPbIqV92AaeXatLxBI9gBaebbnrfifHhDYfgasaacH8akY=wiFfYdH8Gipec8Eeeu0xXdbba9frFj0=OqFfea0dXdd9vqai=hGuQ8kuc9pgc9s8qqaq=dirpe0xb9q8qiLsFr0=vr0=vr0dc8meaabaqaciaacaGaaeqabaqabeGadaaakeaaiiGacqWFbpGCdaqhaaWcbaGaem4AaSMaeiilaWIaemyAaKgabaGaeGymaeJaeiOkaOcaaaaa@3406@(*t*) = (1 - *E*_*S*_)ρk,i1
 MathType@MTEF@5@5@+=feaafiart1ev1aaatCvAUfKttLearuWrP9MDH5MBPbIqV92AaeXatLxBI9gBaebbnrfifHhDYfgasaacH8akY=wiFfYdH8Gipec8Eeeu0xXdbba9frFj0=OqFfea0dXdd9vqai=hGuQ8kuc9pgc9s8qqaq=dirpe0xb9q8qiLsFr0=vr0=vr0dc8meaabaqaciaacaGaaeqabaqabeGadaaakeaaiiGacqWFbpGCdaqhaaWcbaGaem4AaSMaeiilaWIaemyAaKgabaGaeGymaedaaaaa@332A@(*t*), where *E*_*S *_is the reduction in susceptibility to STI due to circumcision.

## Authors' contributions

KD and MCB contributed to the elaboration of the research objectives, development of the mathematical model, computer simulations, statistical analysis of simulated trial data, and manuscript preparation. GPG contributed to the elaboration of the research objectives and manuscript preparation. BRM contributed to the elaboration of the research objectives, statistical analyses of simulated trial data, and manuscript preparation. SM and RCB are co-principal investigators of the UNIM male circumcision study in Kisumu Kenya. They contributed to the elaboration of research objectives, design of the UNIM MC study, statistical analysis of field data to inform the mathematical model, and manuscript preparation. All authors read and approved the final manuscript.
